# *De novo* assembly of the cattle reference genome with single-molecule sequencing

**DOI:** 10.1093/gigascience/giaa021

**Published:** 2020-03-19

**Authors:** Benjamin D Rosen, Derek M Bickhart, Robert D Schnabel, Sergey Koren, Christine G Elsik, Elizabeth Tseng, Troy N Rowan, Wai Y Low, Aleksey Zimin, Christine Couldrey, Richard Hall, Wenli Li, Arang Rhie, Jay Ghurye, Stephanie D McKay, Françoise Thibaud-Nissen, Jinna Hoffman, Brenda M Murdoch, Warren M Snelling, Tara G McDaneld, John A Hammond, John C Schwartz, Wilson Nandolo, Darren E Hagen, Christian Dreischer, Sebastian J Schultheiss, Steven G Schroeder, Adam M Phillippy, John B Cole, Curtis P Van Tassell, George Liu, Timothy P L Smith, Juan F Medrano

**Affiliations:** 1 USDA-ARS, Beltsville, MD, 20705-2350 , Animal Genomics and Improvement Laboratory, USDA-ARS, 10300 Baltimore Ave, Beltsville, MD 20705-2350, USA; 2 Dairy Forage Research Center, USDA-ARS, 1925 Linden Drive, Madison, WI, 53706, USA; 3 Division of Animal Sciences, University of Missouri, 162 Animal Science Research Center, Columbia, MO 65211, USA; 4 Genome Informatics Section, Computational and Statistical Genomics Branch, National Human Genome Research Institute, National Institutes of Health, 9000 Rockville Pike, Bethesda, MD 20892, USA; 5 Pacific Biosciences, 1305 O'Brien Drive, Menlo Park, CA 94025, USA; 6 The Davies Research Centre, School of Animal and Veterinary Sciences, University of Adelaide, Roseworthy, SA 5371, Australia; 7 Johns Hopkins University, Welch Library of Medicine, Ste 105, 1900 E. Monument St., Baltimore, MD 21205, USA; 8 Livestock Improvement Corporation, Private Bag 3016, Hamilton 3240, New Zealand; 9 Department of Computer Science, University of Maryland, 8125 Paint Branch Drive, College Park, MD 20742 USA; 10 Department of Animal and Veterinary Sciences, University of Vermont, Burlington, VT 05405, USA; 11 National Center for Biotechnology Information, National Library of Medicine, National Institutes of Health, Bethesda, MD 20894, USA; 12 Department of Animal and Veterinary Science, University of Idaho, 875 Perimeter Drive MS 2330, Moscow, ID 83844-2330, USA; 13 U.S. Meat Animal Research Center, USDA-ARS, 844 Road 313, Clay Center, NE 68933, USA; 14 The Pirbright Institute, Pirbright, Woking, Surrey, UK; 15 Division of Livestock Sciences, University of Natural Resources and Life Sciences, Gregor Mendel str. 33, A-1180, Vienna, Austria; 16 Animal Science Department, Lilongwe University of Agriculture and Natural Resources, P.O. Box 219, Lilongwe, Malawi; 17 Department of Animal and Food Sciences, Oklahoma State University, 101 Animal Science Building, Stillwater, OK 74078, USA; 18 Computomics GmbH, Christophstr. 32, 72072 Tübingen, Germany; 19 Department of Animal Science, University of California, Davis, One Shields Avenue, Davis, CA 95616, USA

**Keywords:** bovine genome, reference assembly, cattle, Hereford

## Abstract

**Background:**

Major advances in selection progress for cattle have been made following the introduction of genomic tools over the past 10–12 years. These tools depend upon the *Bos taurus* reference genome (UMD3.1.1), which was created using now-outdated technologies and is hindered by a variety of deficiencies and inaccuracies.

**Results:**

We present the new reference genome for cattle, ARS-UCD1.2, based on the same animal as the original to facilitate transfer and interpretation of results obtained from the earlier version, but applying a combination of modern technologies in a *de novo* assembly to increase continuity, accuracy, and completeness. The assembly includes 2.7 Gb and is >250× more continuous than the original assembly, with contig N50 >25 Mb and L50 of 32. We also greatly expanded supporting RNA-based data for annotation that identifies 30,396 total genes (21,039 protein coding). The new reference assembly is accessible in annotated form for public use.

**Conclusions:**

We demonstrate that improved continuity of assembled sequence warrants the adoption of ARS-UCD1.2 as the new cattle reference genome and that increased assembly accuracy will benefit future research on this species.

## Context

**Figure 1:**
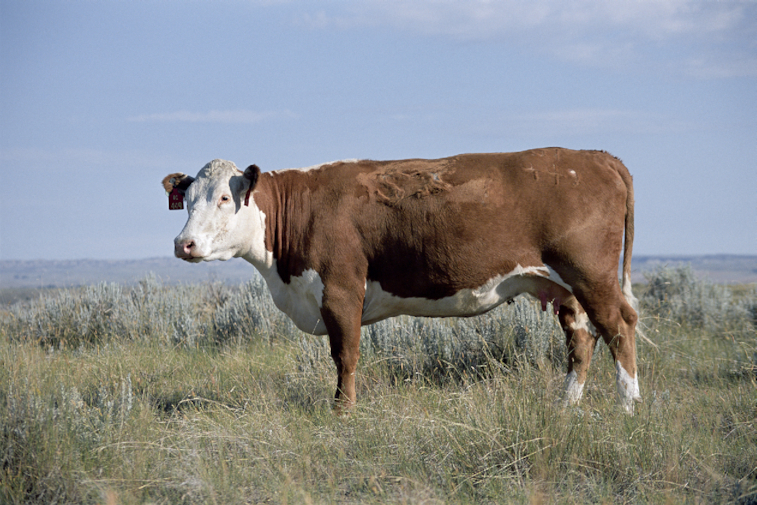
L1 Dominette 0 1449. The line-bred Hereford cow was selected as the original cattle reference animal for her high level of inbreeding.

There are an estimated 1.4 billion domesticated cattle (*Bos taurus*) in the world, being raised primarily for meat and dairy in a diversity of climates and production schemes [1]. This wide diversity of environments has led to the selection of individual breeds of cattle because adaptation for specific needs is required to enhance efficiency and sustainability of production. Despite bottlenecks imposed by breed formation in the relatively recent past, there remains substantial genetic variation within cattle populations that responds to selection for specific traits [2]. Selection progress has been enhanced by the use of genomic tools based on a cattle reference genome [3, 4], especially in dairy cattle in the United States and Europe. The first bovine reference genome was created by a large consortium of researchers and funding institutions, led by the Human Genome Sequencing Center at Baylor College of Medicine. The prevailing methods of the time were improved by the use of inbreeding to decrease the contrast between parental alleles and consequent assembly problems, and by the use of a female to improve coverage of the X chromosome. A Hereford cow, L1 Dominette 0 1449 (Fig. 1), whose sire was also her grandsire and who had an inbreeding coefficient of 0.30, was selected from the U.S. Department of Agriculture (USDA) Agriculture Research Service's Livestock and Range Research Laboratory herd in Miles City, Montana, USA, for creation of the reference assembly [5]. We report a new assembly for the same animal, to provide context for existing data created with the previous reference, but improved by >200-fold in continuity and 10-fold in accuracy. We have also added extensive data to improve the annotation of genes and other genomic features. The new genome and annotation facilitate studies on improving cattle, which is a species of global economic relevance.

## Methods

### Genome sequencing

The original Hereford assembly used blood as the source of DNA, leading to difficulties in assembling specific genomic regions that undergo rearrangement in nucleated blood cells. Therefore, we used high molecular weight genomic DNA extracted from frozen lung tissue as the source for the improved reference, supporting accurate assembly of regions that include important immune function loci. The high molecular weight DNA was extracted and used to construct libraries for single-molecule real-time (SMRT) sequencing as previously described [6]. Libraries were sequenced on a Pacific Biosciences (PacBio) RS II with 318 cells of chemistry P6-C4, yielding 244 Gb (∼80× coverage) of sequence (Table S1) with a mean read length of 20 kb. Additional genomic DNA, also from frozen lung tissue, was used to construct 2 Illumina TruSeq PCR-free 2 × 150 bp paired-end libraries, LIB24773 with a mean insert size of 450 bp and LIB18483 with a mean insert size of 600 bp. The libraries were sequenced on an Illumina NextSeq500, with LIB24773 sequenced on 1 flow cell yielding 111 Gb and LIB18483 sequenced on 2 flow cells yielding 97.6 and 131.3 Gb, respectively (Table S1).

### Assembly, scaffolding, and gap filling

**Figure 2:**
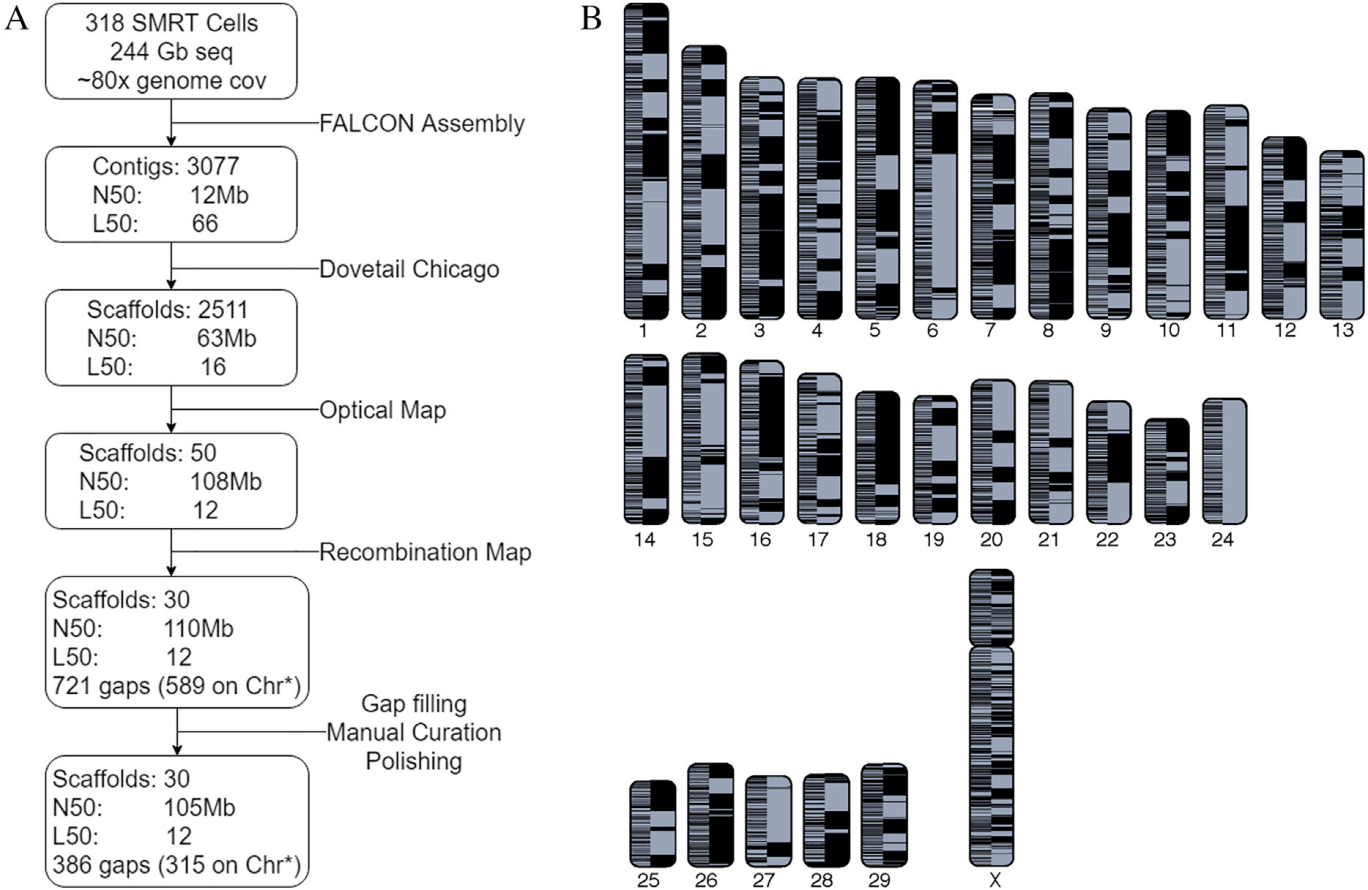
Dominette de novo assembly. A: Assembly pipeline. N50 is the minimum scaffold/contig length needed to cover 50% of the genome; L50 is the number of contigs required to reach N50. B: Cattle chromosomes painted with assembled contigs. A color shift indicates the switch from one contig to the next or the end of an alignment block. The left half of each chromosome shows UMD3.1.1 contigs while the right shows ARS-UCD1.2. To be conservative, contigs were ordered by UMD3.1.1 assembly positions; where there are conflicts in order between ARS-UCD1.2 and UMD3.1.1, the plot displays a color switch in ARS-UCD1.2. Asterisk indicates within scaffolds assigned to chromosomes.

PacBio long reads were assembled using the Falcon *de novo* genome assembler (version 0.4.0) [7]. A length cut-off of 10 kb was used for the initial seed read alignment, and a secondary cut of 8 kb for the pre-assembled reads before layout of the assembly. The assembly resulted in 3,077 primary contigs covering 2.7 Gb with a contig N50 of 12 Mb (Fig. 2). A single round of polishing the assembly was carried out to improve base accuracy [8]. Raw data were mapped back to the assembly using BLASR [9], and a new consensus called with the Quiver algorithm, both carried out using the resequencing pipeline from the SMRT Analysis 3.1.1 software package (Pacific Biosciences, Menlo Park, CA).

Three datasets were used to scaffold contigs: Dovetail Chicago [10], BtOM1.0 optical map [11], and a recombination map developed by Ma et al. [12] (Fig. 2). First, a Chicago library was prepared as described previously [10] from Dominette lung tissue and sequenced on an Illumina HiSeq 2500 to ∼84× coverage (LIB14630, Table S1). The Falcon assembly and Chicago library read pairs were used as input data for HiRise [10], a software pipeline for using Chicago data to scaffold genomes. The separations of Chicago read pairs mapped within contigs were analyzed by HiRise to produce a likelihood model for genomic distance between read pairs, and the model was used to identify putative misjoins and score prospective joins. After scaffolding, long reads were used to close gaps between contigs, resulting in 2,511 scaffolds, with an N50 of 63 Mb and L50 of 16. Next we used the Dominette-derived *B. taurus* optical map BtOM1.0 [11] that spans 2,575.30 Mb and comprises 78 optical contigs to further scaffold the Dovetail assembly. The IrysView v2.5.1 software package (BioNano Genomics, San Diego, CA) was used to map the assembly scaffolds to the optical map contigs. After a manual curation step where false joins and misassembled contigs were detected by inspection of the alignment, IrysView scaffolding reduced the number of scaffolds to 50 while the scaffold L50 decreased to 12 and the scaffold N50 increased to 108 Mb. Finally, ∼54,000 single-nucleotide polymorphism (SNP) markers from the bovine recombination map [12] were used to detect misassemblies and scaffold the 29 acrocentric autosomes [13, 14]. Markers were aligned to the optical map scaffolds with BLAST [15], requiring 98% mapping identity over the full marker sequence length. Only unique mapping SNPs were considered. Scaffolds were broken when ≥2 markers from different linkage groups aligned to them. Pearson correlation coefficients between scaffold marker alignment order and genetic map marker order were used to calculate the most probable scaffold order and orientation. Another round of polishing was undertaken with Arrow with the SMRT Analysis 3.1.1 software package.

Gap filling was first done by aligning 2 Canu (Canu, RRID:SCR_015880) v1.4 [16] assemblies (run with different overlap algorithms implemented within Canu for error correction, MHAP [17] and minimap [18]) to the scaffolded assembly and identifying alignments crossing gaps. A gap was filled if either assembly spanned a gap with >5,000 bp aligning on either side of the gap up to at most 10 bp away from the gap. In the case of a negative gap (i.e., the assemblies had a collapse), both assemblies had to agree on the position and size of the collapse. In total, 171 gaps were closed with this approach. Finally, PBJelly (PBJelly, RRID:SCR_012091) pbsuite v.15.8.24 [19] was used to fill an additional 91 gaps. The closing of gaps between contigs increased the contig N50 from 12 to 21 Mb and reduced the number of gaps in the genome to 459.

### Manual curation

Following gap filling, the assembly was manually curated. To start, we assessed the X chromosome using 2 assemblies produced from MaSuRCA (MaSuRCA, RRID:SCR_010691) [20] error-corrected reads (PacBio corrected with Illumina). The first used Canu v1.4 to assemble the MaSuRCA corrected reads and the other used Celera Assembler [17] version 8.3. MUMmer 3.0 [21] alignments between these 2 assemblies and the gap-filled assembly were used to confirm or revise the order and orientation of X-chromosome contigs as well as to place additional unplaced contigs and scaffolds. Next, the autosomal assembly structure was manually curated and oriented with an independent genetic map UMCLK (Table S2, Supplementary Note). The BLAT (BLAT, RRID:SCR_011919) alignment tool [22] and BWA MEM (BWA, RRID:SCR_010910) [23] were used to map the probe and flanking sequences present on commercially available genotyping assays to identify misassemblies. Assembly gaps, Illumina read-depth coverage, and alignments with dbSNP sequences and flanking sequences were used to refine breakpoints for sequence rearrangements using a combination of custom scripts in iterative fashion [24]. In all, corrections were made to chromosomes 1, 2, 5–12, 16, 18–21, 23, 26, 27, and X. PBJelly was run on the curated assembly to close remaining gaps. The number of gaps decreased from 459 to 386, indicating that our manual curation correctly oriented contigs such that PBJelly could now fill an additional 73 gaps that could not previously be filled. The remaining gaps represent regions where either the gap is too large for our PacBio reads to span, read coverage is low or missing, or there is a remaining misassembly. The contig N50 also increased again from 21 to 26 Mb. Polishing of the assembly proceeded through 1 iteration of Arrow with all the raw PacBio reads followed by polishing with short Illumina reads (SRR2226514 and SRR2226524 as well as LIB24773 and 1 run, 97.6 Gb, of LIB18483) using Pilon (Pilon, RRID:SCR_014731) v1.22 [25] with the parameters “–diploid –fix indels –nostrays.” The final version of the genome (ARS-UCD1.2) contains 2,628,394,923 bp on the 30 chromosomes (Fig. 2b) with an additional 87.5 Mb of unplaced sequence and is available from NCBI under the accession GCF_0 022 63795.1.

### RNA sequencing

The Iso-Seq method for sequencing full-length transcripts was developed by PacBio during the same time period as the genome assembly. We therefore used this technique to improve characterization of transcript isoforms expressed in cattle tissues using a diverse set of tissues collected from L1 Dominette 0 1449 upon euthanasia. The data were collected using an early version of the Iso-Seq library protocol [26] as suggested by PacBio. Briefly, RNA was extracted from each tissue using Trizol reagent as directed (Thermo Fisher). Then 2 μg of RNA were selected for PolyA tails and converted into complementary DNA (cDNA) using the SMARTer PCR cDNA Synthesis Kit (Clontech). The cDNA was amplified in bulk with 12–14 rounds of PCR in 8 separate reactions, then pooled and size-selected into 1–2, 2–3, and 3–6 kb fractions using the BluePippin instrument (Sage Science). Each size fraction was separately re-amplified in 8 additional reactions of 11 PCR cycles. The products for each size fraction amplification were pooled and purified using AMPure PB beads (Pacific Biosciences) as directed, and converted to SMRTbell libraries using the Template Prep Kit v1.0 (PacBio) as directed. Iso-Seq was conducted for 22 tissues including abomasum, aorta, atrium, cerebral cortex, duodenum, hypothalamus, jejunum, liver, longissimus dorsi muscle, lung, lymph node, mammary gland, medulla oblongata, omasum, reticulum, rumen, subcutaneous fat, temporal cortex, thalamus, uterine myometrium, and ventricle from the reference cow, as well as the testis of her sire. The size fractions were sequenced in either 4 (for the smaller 2 fractions) or 5 (for the largest fraction) SMRTcells on the RS II instrument. Isoforms were identified using the Cupcake ToFU pipeline [27] without using a reference genome.

Short-read–based RNA-seq data derived from tissues of Dominette were available in the GenBank database because her tissues have been a freely distributed resource for the research community. To complement and extend these data and to ensure that the tissues used for Iso-Seq were also represented by RNA-seq data for quantitative analysis and confirmation of isoforms observed in Iso-Seq, we generated additional data, avoiding overlap with existing public data. Specifically, the TruSeq stranded mRNA LT kit (Illumina, Inc.) was used as directed to create RNA-seq libraries, which were sequenced to ≥30 million reads for each tissue sample. The Dominette tissues that were sequenced in this study include abomasum, anterior pituitary, aorta, atrium, bone marrow, cerebellum, duodenum, frontal cortex, hypothalamus, KPH fat (internal organ fat taken from the covering on the kidney capsule), lung, lymph node, mammary gland (lactating), medulla oblongata, nasal mucosa, omasum, reticulum, rumen, subcutaneous fat, temporal cortex, thalamus, uterine myometrium, and ventricle. RNA-seq libraries were also sequenced from the testis of her sire. All public datasets, and the newly sequenced RNA-seq and Iso-Seq datasets, were used to annotate the assembly, to improve the representation of low-abundance and tissue-specific transcripts, and to properly annotate potential tissue-specific isoforms of each gene.

### Annotation

The NCBI Eukaryotic Genome Annotation Pipeline was used to annotate genes, transcripts, proteins, and other genomic features on ARS-UCD1.2. Nearly 13 billion RNA-seq reads from >50 tissues and 553,798 consensus Iso-Seq reads from 23 tissues were retrieved from SRA (Table S1) and aligned to the masked genome, along with 12,472 known RefSeq transcripts, 19,820 GenBank transcripts, and 1,583,270 expressed sequence tags, using BLAST [15] followed by Splign [28]. The set of proteins aligned to the masked genome consisted of 13,381 RefSeq proteins and 16,371 GenBank proteins from cattle and 50,089 RefSeq proteins from human. The gene models’ structures and boundaries were primarily derived from these alignments. Where alignments did not define a complete model but the coding propensity of the region was sufficiently high, *ab initio* extension or joining/filling of partial open reading frames in compatible frame was performed by Gnomon [29], using a hidden Markov model trained on cattle. Transfer RNAs (tRNAs) were predicted with tRNAscan-SE (tRNAscan-SE, RRID:SCR_010835) 1.23 [30] and small non-coding RNAs were predicted by searching the RFAM 12.0 HMMs for eukaryotes using cmsearch from the Infernal (Infernal, RRID:SCR_011809) package [31]. The annotation of the ARS-UCD1.2 assembly, Annotation Release 106 (AR 106 [32]), resulted in 21,039 protein-coding genes, 9,357 non-coding genes, and 4,569 pseudogenes.

The respective quality of the UMD3.1.1 annotation (Annotation Release 105 [AR 105] [33]) and AR 106 was evaluated by aligning the annotated proteins of each release to the UniProtKB/SwissProt proteins available in Entrez Protein (returned by the Entrez query srcdb_swiss_prot[properties] AND eukaryotes[orgn] on 29 July 2019) using BlastP. For each protein-coding gene, the protein isoform with the best alignment based on score (or in case of a tie, based on alignment length, percent coverage, or subject protein length) was chosen as the isoform representative of the gene. The counts of protein-coding genes in AR 105 and AR 106 with representative isoforms covering ≥95% of the length of the UniProtKB/SwissProt proteins were then compared.

## Data Validation and Quality Control

### Quality assessment

**Figure 3:**
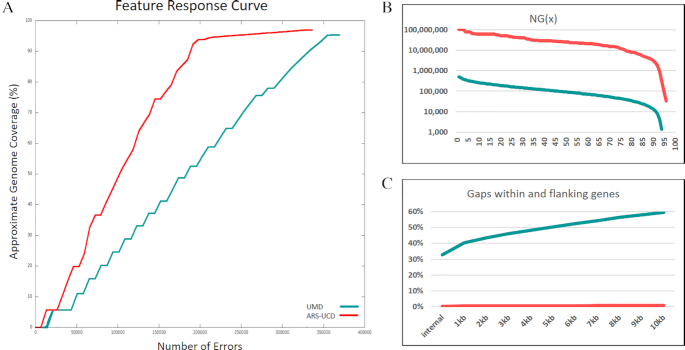
Assembly assessments computed for ARS-UCD1.2 and UMD3.1.1. A, Feature response curves computed for ARS-UCD1.2 and UMD3.1.1. B, Calculated contig NGx (minimum contig length needed to cover x% of the genome calculated on a fixed genome size of 2.8 Gb) showing a 280-fold increase of ARS-UCD1.2 in comparison with UMD3.1.1. C, The percentage of gaps in gene-flanking regions is reduced from 33% to 0.3% in ARS-UCD1.2 in comparison with UMD3.1.1.

To assess the error profile of our assembly and compare it to the previous reference, UMD3.1.1 [34] (NCBI accession GCF_0 00003055.5), long- and short-read sequences from Dominette were aligned to both assemblies. Short-read BWA alignments of LIB18483 sequences not used for polishing were evaluated from feature response curves computed with FRCbam [35] (Fig. 3A). The total number of erroneous features in ARS-UCD1.2 decreased by >20% compared with UMD3.1.1 (Table 1). Errors on the chromosome scaffolds exhibited a >40% reduction in error features compared with UMD3.1.1, suggesting that ARS-UCD1.2 chromosomes were better representative of the individual sequenced. The error classes most prevalent on the ARS-UCD1.2 unplaced sequences compared to the chromosomes were HIGH COV PE, HIGH NORM COV PE, and HIGH SPAN PE with unplaced sequences accounting for 73%, 80%, and 65% of the errors in each class, respectively. The increased percentage of HIGH COV PE and HIGH NORM COV PE errors indicates that many of the unplaced sequences are over-assembled or collapsed, while HIGH SPAN PE errors would be expected because the majority of the 2,181 unplaced sequences are fragmented. The same short-read alignments were also used to estimate the quality value of the assembly, with ARS-UCD1.2 scoring 48.67 and UMD3.1.1 37.98, which correspond to a per-base error rate of 1.58 × 10^−5^ and 1.59 × 10^−4^, respectively, or an order-of-magnitude improvement in accuracy. This was calculated from the number of non-matching base calls from FreeBayes (FreeBayes, RRID:SCR_010761 ) [36] as previously described [6]. UMD3.1.1’s lower per-base accuracy resulted from the large number of gaps in the assembly, the larger proportion of unplaced contigs, and the incomplete resolution of larger repetitive regions.

**Table 1:** Assembly quality score value statistics and structural inconsistencies measured between ARS-UCD1.2 and UMD3.1.1 using Dominette whole-genome sequencing reads

Major category	Subcategory	ARS-UCD1.2	UMD3.1.1	Description
QV		48.67	37.98	Quality value estimate (Phred-scale)
FRCbam				
	COMPR PE	37,309 (30,643)^1^	54,602 (52,606)	Areas with low CE statistics
	STRECH PE	37,255 (22,741)	35,766 (35,299)	Areas with high CE statistics
	HIGH COV PE	7,166 (1,970)	7,711 (6,331)	High read coverage areas (all aligned reads)
	HIGH NORM COV PE	5,641 (1,125)	7,109 (5,778)	High paired-read coverage areas (only properly aligned pairs)
	HIGH OUTIE PE	139 (102)	2,108 (2,108)	Regions with high numbers of misoriented or distant pairs
	HIGH SINGLE PE	60 (53)	1,258 (1,256)	Regions with high numbers of unmapped pairs
	HIGH SPAN PE	4,882 (1,687)	4,172 (3,582)	Regions with high numbers of pairs that map to different scaffolds
	LOW COV PE	43,370 (36,062)	57,176 (56,648)	Low read coverage areas (all aligned reads)
	LOW NORM COV PE	42,067 (34,592)	60,560 (59,926)	Low paired-end coverage areas (only properly aligned pairs)
	Total features	177,889 (128,975)	230,462 (223,534)	All erroneous features
Sniffles^2^				
	DEL	188	10,504	Deletions
	DUP	16	728	Duplications
	INS	106	4,911	Insertions
	INV	34	2,675	Inversions
	Total SVs	344	18,818	All structural variants

1 Numbers in parentheses indicate the errors in placed chromosome scaffolds only.

2 Sniffles structural variant (SV) calls were generated using long reads aligned to the whole assembly. CE: compression/expansion; QV: quality value.

As a measure of the completeness of the assemblies and to define the chromosome ends, we identified centromeric [24] and telomeric [37] repeats (Table S3). For the 29 acrocentric autosomes, we identified the expected centromeric and telomeric repeats on 9 ARS-UCD1.2 chromosomes (5, 6, 8, 10, 13, 14, 16–18) whereas no UMD3.1.1 chromosomes contained both, mainly owing to a relative lack of telomeric repeats in the assembly. ARS-UCD1.2 chromosomes 3, 20, and 22 are missing both chromosome ends, while chromosomes 1, 9, 10, and 15 erroneously contain centromeric repeats at both ends. Finally, the metacentric X chromosome only has telomeric repeats at 1 end and no centromeric repeats. Telomeric repeats were only identified on UMD3.1.1 chromosome 20, centromeric repeats are found on the proper end of 22 autosomes (missing on 6, 7, 20–22, 27, and 28), and the X chromosome contains centromeric repeats. All chromosomes also contain centromeric repeats dispersed throughout, so it is difficult to determine whether the X centromere is properly placed. Centromeric repeat regions at the start of ARS-UCD1.2 chromosome scaffolds were >2-fold larger than their counterparts in the UMD3.1 reference (Figure S1). To further assess the structural integrity of both assemblies, we used Sniffles [38] to evaluate the concordance of long reads from Dominette on both assemblies. All SV classes showed sharp declines in prevalence in ARS-UCD1.2 vs UMD3.1.1 (Table 1). Deletions, duplications, insertions, and inversions all declined by ≥98%.

### Improved contiguity

A key measure of improvement over the previous reference is the increase in the contiguity of the genome (Fig. 2). The 30 cattle chromosomes are now composed of 345 contigs compared to 72,264 contigs in the UMD3.1.1 assembly. This represents a 280-fold increase in the contig NG50 (N50 calculated from a fixed 2.8-Gb genome size), from 0.092 to 25.8 Mb (Fig. 3b), and a 209-fold increase in sequence continuity. The 345 contigs in ARS-UCD1.2 equate to 315 gaps in the chromosomes vs 72,234 on UMD3.1.1. We demonstrated the impact of higher contiguity on the mapping of existing datasets by aligning the currently available 14,473 known cattle RefSeq transcripts (a manually curated set of transcript accessions prefixed with NM_ and NR_) to both ARS-UCD1.2 and UMD3.1.1. We found that the transcripts aligned more cleanly to ARS-UCD1.2 than to UMD3.1.1 (Table 2). The number of transcripts for which the best alignment covered <95% of the CDS decreased from 734 on UMD3.1.1 to only 37 for ARS-UCD1.2. Moreover, the alignment of 219 transcripts was split across 2 or more genomic sequences of UMD3.1.1 compared with only 9 for ARS-UCD1.2. Although a greater number of transcripts failed to align to ARS-UCD1.2, this difference is made up of transcripts from Y-linked genes (Table S4). The presence of Y-linked genes in the UMD3.1.1 assembly is likely due to Y chromosome contamination from the inclusion of sequence from a bacterial artificial chromosome library prepared from Dominette's sire [34, 39]. Because ARS-UCD1.2 is derived from an XX female and does not contain the Y chromosome, we recommend the inclusion of an independently assembled Y chromosome prior to analysis as is being done by the 1000 Bull Genomes Project [40].

**Table 2:** Splign alignment of RefSeq transcripts to ARS-UCD1.2 and UMD3.1.1

Parameter	ARS-UCD1.2	UMD3.1.1
Accession	GCF_0 022 63795.1	GCF_0 00003055.5
No. of sequences retrieved from Entrez	14,473	14,473
No. of sequences not aligning^1^	19 (12)	13 (12)
No. of sequences whose best alignments span multiple loci (split genes)	9	219
No. of sequences with CDS coverage <95%	37	734

1 Neither assembly includes a Y chromosome, yet 7 transcripts (6 not aligning to only ARS-UCD1.2 and 1 not aligning to both) are from Y-linked genes. Totals excluding Y-linked genes in parentheses.

### Annotation comparison

The ARS-UCD1.2 assembly annotation (AR 106) generated by NCBI was compared with the UMD3.1.1 annotation (AR 105). Approximately two-thirds of the genes (85% of protein-coding genes) are identical or nearly identical (with a support score, derived from a combination of matching exon boundaries and sequence overlap, of ≥0.66, on a scale of 0 to 1, on both sides of the comparison) between the 2 datasets (Table S5). More than 90% of the novel genes (19% of total genes) in AR 106 were non-coding genes, owing in part to the addition of a module for the prediction of short non-coding genes based on RFAM models to the annotation pipeline after AR 105 was produced. The number of protein-coding genes with ≥1 isoform covering 95% of the length of a UniProt/SwissProtKB protein is 17,810 (85% of protein-coding genes) for AR 106 versus 16,956 (80%) for AR 105, suggesting that the protein models predicted in AR 106 are generally more complete than in AR 105.

These improvements in the annotation are partly due to the availability of more and longer transcript evidence for gene prediction (Iso-Seq in particular), but it is clear that uncertainty of placement and orientation of sequence across gaps has a large impact on gene annotation. Of the 21,039 genes annotated in ARS-UCD1.2, 69 (0.3%) have gaps within introns compared to 6,949 (33%) of annotated UMD3.1.1 genes (Fig. 3c). Considering the potential impact of regulatory elements flanking genes, it is also important to note that almost 60% of UMD3.1.1 genes have gaps within 10 kb while that percentage decreases to <1% in ARS-UCD1.2.

ARS-UCD1.2 also represents an improvement in base accuracy over UMD3.1.1 that is measurable in the annotation. High rates of sequencing error can disrupt the prediction of open reading frames and lead to truncated gene models or the erroneous calling of non-coding genes or pseudogenes instead of protein-coding genes. The NCBI annotation process attempts to compensate for this problem by producing a “corrected” model (with name prefixed with LOW QUALITY) containing a difference with the genome sequence, when protein alignments suggest that there is an erroneous indel in the genome. The number of such “corrected” models decreased by 44% from 1,828 in UMD3.1.1/AR 105 to 1,027 in ARS-UCD1.2/AR 106.

## Conclusions

This assembly represents a 200-fold improvement in sequence continuity and a 10-fold improvement in per-base accuracy over previous cattle assemblies. The assignment of megabase-length contigs to full chromosome scaffolds provides additional certainty in gene and genetic marker positions, which will influence marker-assisted selection and basic research. The assembly was selected as the reference genome for taurine cattle by the US genomic evaluation system in December 2018 [41] and the 37 partner institutions of the 1000 Bull Genomes Project for the run7 variant calls distributed globally in June 2019 [40]. We demonstrate that assembly improvements warranted adoption by these projects and that increased assembly accuracy will benefit future genetics research on this species.

## Availability of Supporting Data and Materials

Accession numbers for raw sequencing reads and assemblies can be found in Table S1. Supporting data are also available through a GigaDB dataset [42].

## Additional Files

**Table S1:** Sequencing resources

**Table S2:** UMCLK genetic map

**Table S3:** Centromeric and telomeric repeats

**Table S4:** RefSeq transcripts not aligning to assemblies

**Table S5:** ARS-UCD1.2 vs UMD3.1.1 annotation comparison

**Figure S1:** Average centromeric repeat regions. Centromeric satellite regions identified by RepeatMasker in the ARS-UCD1.2 (ARSUCD) and UMD3.1.1 (UMD3) assemblies were merged if they overlapped by 1 bp. Histogram bars show the mean length of these regions that are within the first 500 kb of a chromosome scaffold's starting base (CHRSTART), within unplaced scaffolds (UNPLACED), or in the middle of the chromosome scaffolds (CHRSCAFF). Error bars represent the 95% confidence interval (2 standard errors from the mean) of centromere lengths in each category.

**Supplemental Note**. UMCLK genetic map

## Abbreviations

AR: annotation release; BLASR: Basic Local Alignment with Successive Refinement; BLAST: Basic Local Alignment Search Tool; bp: base pairs; BWA: Burrows-Wheeler Aligner; cDNA: complementary DNA; dbSNP: Database of Single Nucleotide Polymorphisms; Gb: gigabase pairs; kb: kilobase pairs; Mb: megabase pairs; mRNA: mesenger RNA; NCBI: National Center for Biotechnology Information; PacBio: Pacific Biosciences; PE: paired end; RefSeq: NCBI Reference Sequence Database; RNA-seq: high-throughput short-read messenger RNA sequencing; SMRT: single-molecule real-time; SNP: single-nucleotide polymorphism; SRA: Sequence Read Archive; SV: structural variant; tRNA: transfer RNA; USDA: United States Department of Agriculture.

## Competing Interests

R.H. and E.T. are employed by Pacific Biosciences; all other authors declare that they have no competing interests.

## Funding

Sequence generation was supported by USDA/NRSP-8 Animal Genome, USDA-ARS Meat Animal Research Center, Neogen, and Zoetis. B.D.R., S.G.S., J.B.C., C.P.V.T., and G.L. were supported by USDA CRIS 8042-31000-001-00-D. J.B.C. was supported by USDA CRIS 8042-31000-002-00-D. D.M.B. and W.L. were supported in part by USDA CRIS 5090-31000-026-00-D. D.M.B. was also supported in part by USDA NIFA grant 5090-31000-026-06-I. W.M.S., T.G.M., T.P.L.S. were supported by USDA CRIS 3040-31000-100-00-D. R.D.S. and T.N.R. were supported in part by USDA NIFA 2016-68004-24827. R.D.S. was also supported in part by USDA NIFA 2013-67015-21202, 2015-67015-23183, NIH 1R01HD084353-01A1, and USDA Hatch MO-HAAS0001. J.H. and F.T.N. were supported by the Intramural Research Program of the National Library of Medicine, National Institutes of Health. J.A.H. and J.C.S. were supported by funding from the UKRI-BBSRC awards BB/M027155/1, BBS/E/I/00007035, BBS/E/I/00007038, and BBS/E/I/00007039. S.K., A.R., and A.M.P. were supported by the Intramural Research Program of the National Human Genome Research Institute, National Institutes of Health. A.R. was also supported by the Korean Visiting Scientist Training Award (KVSTA) through the Korea Health Industry Development Institute (KHIDI), funded by the Ministry of Health & Welfare (HI17C2098). This work utilized the computational resources of the NIH HPC Biowulf cluster (https://hpc.nih.gov). Mention of trade names or commercial products in this article is solely for the purpose of providing specific information and does not imply recommendation or endorsement by the U.S. Department of Agriculture.

## Authors' Contributions

T.P.L.S., J.F.M., C.P.V.T., R.D.S., D.M.B., and B.D.R. conceived, initiated, and managed the project. T.P.L.S. and J.F.M. were responsible for DNA and RNA sequence data production. B.D.R., D.M.B., R.D.S., S.J.S., C.D., A.Z., R.H., J.G., A.R., S.K., and A.M.P. performed assembly and associated tasks. T.N.R., W.Y.L., C.C., W.L., S.D.M., B.M.M., W.M.S., J.A.H., J.C.S., W.N., S.G.S., J.B.C., G.L., and C.P.V.T. performed quality control and/or contributed additional analyses. C.G.E., T.G.M., and E.T. performed RNA analyses. F.T.N. and J.H. performed annotation and managed public presentation of the assembly files. All authors read and edited the manuscript.

## Supplementary Material

giaa021_GIGA-D-19-00331_Original_Submission

giaa021_GIGA-D-19-00331_Revision_1

giaa021_Response-to-Reviewer_Comments_Original_Submission

giaa021_Reviewer_1_Report_Original_Submission Paul Stothard -- 10/7/2019 Reviewed

giaa021_Reviewer_2_Report_Original_Submission Alan Archibald -- 10/22/2019 Reviewed

giaa021_Supplemental_Data
