# Supplementary material for: De novo assembly of the cattle reference genome with single-molecule sequencing
Source: Gigascience. 2020 Mar 19;9(3):giaa021. doi: 10.1093/gigascience/giaa021 (PMC7081964; doi:10.1093/gigascience/giaa021)
Supplement: giaa021_GIGA-D-19-00331_Original_Submission [file giaa021_giga-d-19-00331_original_submission.pdf]

GigaScience

De novo assembly of the cattle reference genome with single-molecule sequencing

--Manuscript Draft--

**Manuscript Number:**	GIGA-D-19-00331
**Full Title:**	De novo assembly of the cattle reference genome with single-molecule sequencing
**Article Type:**	Data Note

Funding Information:

USDA/NRSP8 Animal Genome	Not applicable
USDA ARS Meat Animal Research Center	Not applicable
Neogen	Not applicable
Zoetis	Not applicable
Agricultural Research Service (8042-31000-001-00-D)	Not applicable
Agricultural Research Service (8042-31000-002-00-D)	Not applicable
Agricultural Research Service (5090-31000-026-00-D)	Not applicable
National Institute of Food and Agriculture (5090-31000-026-06-I)	Dr Derek M Bickhart
Agricultural Research Service (3040-31000-100-00-D)	Not applicable
National Institute of Food and Agriculture (2016-68004-24827,2013-67015-21202, 2015-67015-23183)	Dr Robert D Schnabel
National Institutes of Health (1R01HD084353-01A1)	Dr Robert D Schnabel
USDA Hatch (MO-HAAS0001)	Dr Robert D Schnabel
U.S. National Library of Medicine	Not applicable
Biotechnology and Biological Sciences Research Council (BB/M027155/1, BBS/E/I/00007035, BBS/E/I/00007038 and BBS/E/I/00007039)	Dr John A Hammond
National Human Genome Research Institute	Not applicable

Abstract:

Major advances in selection progress for cattle have been made following the introduction of genomic tools over the past 10-12 years. These tools depend upon the Bos taurus reference genome (UMD3.1.1), which was created using now-outdated technologies and suffers from a variety of deficiencies and inaccuracies. We present the new reference genome for cattle, ARS-UCD1.2, based on the same animal as the original to facilitate transfer and interpretation of results obtained from the earlier version, but applying a combination of modern technologies in a de novo assembly to increase continuity, accuracy, and completeness. The assembly includes 2.7 Gb, and is >250x more continuous than the original assembly, with contig N50 >25 Mb and L50 of 32. We also greatly expanded supporting RNA-based data for annotation that identifies 30,396 total genes (21,039 protein coding). The new reference assembly is accessible in annotated form for public use.

**Corresponding Author:**	Benjamin D Rosen<br><br>UNITED STATES
**Corresponding Author Secondary Information:**	
**Corresponding Author's Institution:**	
**Corresponding Author's Secondary**	

Powered by Editorial Manager® and ProduXion Manager® from Aries Systems Corporation

**Institution:**	
**First Author:**	Benjamin D Rosen
**First Author Secondary Information:**	
**Order of Authors:**	Benjamin D Rosen
	Derek M Bickhart
	Robert D Schnabel
	Sergey Koren
	Christine G Elsik
	Elizabeth Tseng
	Troy N Rowan
	Wai Y Low
	Aleksey Zimin
	Christine Couldrey
	Richard Hall
	Wenli Li
	Arang Rhie
	Jay Ghurye
	Stephanie D McKay
	Francoise Thibaud-Nissen
	Jinna Hoffman
	Brenda M Murdoch
	Warren M Snelling
	Tara G McDaneld
	John A Hammond
	John C Schwartz
	Wilson Nandolo
	Darren E Hagen
	Christian Dreischer
	Sebastian J Schultheiss
	Steven G Schroeder
	Adam M Phillippy
	John B Cole
	Curtis P Van Tassell
	George Liu
	Timothy P.L. Smith
	Juan F Medrano
**Order of Authors Secondary Information:**	
**Additional Information:**	
**Question**	**Response**

Are you submitting this manuscript to a special series or article collection?	No
**Experimental design and statistics**<br><br>Full details of the experimental design and statistical methods used should be given in the Methods section, as detailed in our Minimum Standards Reporting Checklist. Information essential to interpreting the data presented should be made available in the figure legends.<br><br>Have you included all the information requested in your manuscript?	Yes
**Resources**<br><br>A description of all resources used, including antibodies, cell lines, animals and software tools, with enough information to allow them to be uniquely identified, should be included in the Methods section. Authors are strongly encouraged to cite Research Resource Identifiers (RRIDs) for antibodies, model organisms and tools, where possible.<br><br>Have you included the information requested as detailed in our Minimum Standards Reporting Checklist?	Yes
**Availability of data and materials**<br><br>All datasets and code on which the conclusions of the paper rely must be either included in your submission or deposited in publicly available repositories (where available and ethically appropriate), referencing such data using a unique identifier in the references and in the "Availability of Data and Materials" section of your manuscript.<br><br>Have you have met the above requirement as detailed in our Minimum	Yes

Standards Reporting Checklist?	

Powered by Editorial Manager® and ProduXion Manager® from Aries Systems Corporation

Manuscript

Click here to download Manuscript ARS-UCD-DataNote_submission_unlinked.docx

GIGASCIENCE, DATA NOTE

De novo assembly of the cattle reference genome with single-molecule sequencing

Benjamin D. Rosen[1†*] ben.rosen@usda.gov (Corresponding Author), Derek M. Bickhart[2†] derek.bickhart@usda.gov, Robert D. Schnabel[3†] schnabelr@missouri.edu, Sergey Koren[4] sergey.koren@nih.gov, Christine G. Elsik[3] elsikc@missouri.edu, Elizabeth Tseng[5] etseng@pacificbiosciences.com, Troy N. Rowan[3] tnr343@mail.missouri.edu, Wai Y. Low[6] wai.low@adelaide.edu.au, Aleksey Zimin[7,8] alekseyz@jhu.edu, Christine Couldrey[9] christine.couldrey@lic.co.nz, Richard Hall[5] rhall@pacificbiosciences.com, Wenli Li[2] wenli.li@usda.gov, Arang Rhie[4] rhiea@nih.gov, Jay Ghurye[10] jayg@cs.umd.edu, Stephanie D. McKay[11] stephanie.mckay@uvm.edu, Françoise Thibaud-Nissen[12] thibaudf@ncbi.nlm.nih.gov, Jinna Hoffman[12] jinna.choi@nih.gov, Brenda M. Murdoch[13] bmurdoch@uidaho.edu, Warren M. Snelling[14] warren.snelling@usda.gov, Tara G. McDaneld[14] tara.mcdaneld@usda.gov, John A. Hammond[15] john.hammond@pirbright.ac.uk, John C. Schwartz[15] john.schwartz@pirbright.ac.uk, Wilson Nandolo[16,17] wilsonandolo@gmail.com, Darren E. Hagen[18] darren.hagen@okstate.edu Christian Dreischer[19] christian.dreischer@computomics.com, Sebastian J Schultheiss[19] sebastian.schultheiss@computomics.com, Steven G. Schroeder[1] steven.schroeder@usda.gov, Adam M. Phillippy[4] adam.phillippy@nih.gov, John B. Cole[1] john.cole@usda.gov, Curtis P. Van Tassell[1] curt.vantassell@usda.gov, George Liu[1] george.liu@usda.gov, Timothy P.L. Smith[14*] tim.smith2@usda.gov (Corresponding Author), Juan F. Medrano[20] jfmedrano@ucdavis.edu

Affiliations

[1]Animal Genomics and Improvement Laboratory, USDA-ARS, Beltsville, MD, USA

[2]Dairy Forage Research Center, USDA-ARS, Madison, WI, USA

[3]University of Missouri, Columbia, MO, USA

[4]Genome Informatics Section, Computational and Statistical Genomics Branch, National Human Genome Research Institute, National Institutes of Health, Bethesda, MD, USA

[5]Pacific Biosciences, Menlo Park, CA, USA

[6]The Davies Research Centre, University of Adelaide, Roseworthy, Australia

[7]Johns Hopkins School of Medicine, Baltimore, MD, USA

[8]Johns Hopkins University, Baltimore, MD, USA

[9]Livestock Improvement Corporation, Hamilton, New Zealand

[10] University of Maryland, College Park, MD, USA

[11]University of Vermont, Burlington, VT, USA

[12]National Center for Biotechnology Information, National Library of Medicine, National Institutes of Health, Bethesda, MD, USA

[13]University of Idaho, Moscow, ID, USA

[14]U.S. Meat Animal Research Center, USDA-ARS, Clay Center, NE, USA

[15]The Pirbright Institute, Woking, UK

[16]University of Natural Resources and Life Sciences, Vienna, Austria

[17]Lilongwe University of Agriculture and Natural Resources, Lilongwe, Malawi

[18]Oklahoma State University, Stillwater, OK, USA

[19]Computomics GmbH, Tuebingen, Germany

[20]University of California, Davis, CA, USA

[†]These authors contributed equally to this work

*Correspondence: ben.rosen@usda.gov, tim.smith2@usda.gov

Abstract

Major advances in selection progress for cattle have been made following the introduction of genomic tools over the past 10-12 years. These tools depend upon the *Bos taurus* reference genome (UMD3.1.1), which was created using now-outdated technologies and suffers from a variety of deficiencies and inaccuracies. We present the new reference genome for cattle, ARS-UCD1.2, based on the same animal as the original to facilitate transfer and interpretation of results obtained from the earlier version, but applying a combination of modern technologies in a *de novo* assembly to increase continuity, accuracy, and completeness. The assembly includes 2.7 Gb, and is >250x more continuous than the original assembly, with contig N50 >25 Mb and L50 of 32. We also greatly expanded supporting RNA-based data for annotation that identifies 30,396 total genes (21,039 protein coding). The new reference assembly is accessible in annotated form for public use.

Keywords

Bovine genome, reference assembly, cattle, Hereford

Data Description

Context

There are an estimated 1.4 billion domesticated cattle (*Bos taurus*) in the world, being raised primarily for meat and dairy in a diversity of climates and production schemes[1]. This wide diversity of environments has led to the selection of individual breeds of cattle, as adaptation for specific needs is required to enhance efficiency and sustainability of production. Despite bottlenecks imposed by breed formation in the relatively recent past, there remains substantial

genetic variation within cattle populations that responds to selection for specific traits[2]. Selection progress has been enhanced by the use of genomic tools based on a cattle reference genome[3,4], especially in dairy cattle in the U.S. and Europe. The first bovine reference genome was created by a large consortium of researchers and funding institutions, led by the Human Genome Sequencing Center at Baylor College of Medicine. The prevailing methods of the time were improved by the use of inbreeding to decrease the contrast between parental alleles and consequent assembly problems, and by the use of a female to improve coverage of the X chromosome. A Hereford cow, L1 Dominette 01449, whose sire was also her grandsire and who had an inbreeding coefficient of 0.30, was selected from the USDA Agriculture Research Service's Livestock and Range Research Laboratory herd in Miles City, Montana, USA for creation of the reference assembly[5]. We report a new assembly for the same animal, to provide context for existing data created with the previous reference, but improved by over 200-fold in continuity and accuracy. We have also added extensive data to improve the annotation of genes and other genomic features. The new genome and annotation facilitate studies on improving cattle, which is a species of global economic relevance.

Methods

a) Genome sequencing

The original Hereford assembly used blood as the source of DNA, leading to difficulties in assembling specific genomic regions that undergo rearrangement in nucleated blood cells. Therefore, we used high molecular weight (HMW) genomic DNA extracted from frozen lung tissue as the source for the improved reference, supporting accurate assembly of regions that include important immune function loci. The HMW DNA was extracted and used to construct

libraries for SMRT sequencing as previously described[6]. Libraries were sequenced on a PacBio RS II with 318 cells of chemistry P6-C4 yielding 244 Gb (~80x coverage) of sequence (Table S1) with an average read length of 20 kb. Additional genomic DNA, also from frozen lung tissue, was used to construct two Illumina TruSeq PCR-free 2x150 bp paired-end libraries, LIB24773 with an average insert size of 450 bp and LIB18483 with an average insert size of 600 bp. The libraries were sequenced on an Illumina NextSeq500 with LIB24773 sequenced on one flow cell yielding 111 Gb and LIB18483 sequenced on two flow cells yielding 97.6 Gb and 131.3 Gb, respectively (Table S1).

b) Assembly, scaffolding and gap filling

PacBio long reads were assembled using the Falcon *de novo* genome assembler (version 0.4.0)[7]. A length cutoff of 10 kb was used for the initial seed read alignment, and a secondary cut of 8 kb for the pre-assembled reads before layout of the assembly. The assembly resulted in 3077 primary contigs covering 2.7 Gb with a contig N50 of 12 Mb (Figure 1). A single round of polishing the assembly was carried out to improve base accuracy[8]. Raw data was mapped back to the assembly using blasr[9], and a new consensus called with the Quiver algorithm, both carried out using the resequencing pipeline from the SMRT Analysis 3.1.1 software package (Pacific Biosciences, Menlo Park, CA).

Scaffolding proceeded through the application of three data sets: Dovetail Chicago[10], BtOM1.0 optical map[11], and a recombination map developed by Ma *et al*.[12] (Figure 1). First, a Chicago library was prepared as described previously[10] and sequenced on an Illumina HiSeq 2500 to approximately 84x coverage (Table S1). The Falcon assembly and Chicago library read pairs were used as input data for HiRise[10], a software pipeline for using Chicago data to scaffold genomes. The separations of Chicago read pairs mapped within contigs were analyzed by HiRise to produce a likelihood model for genomic distance between read pairs, and the model was used to identify putative misjoins and score prospective joins. After scaffolding, long reads were used to close gaps between contigs resulting in 2511 scaffolds with an N50 of 63 Mb and L50 of 16. Next we used the *Bos taurus* optical map BtOM1.0[11] that spans 2,575,30 Mb and comprises 78 optical contigs to further scaffold the Dovetail assembly. The IrysView v2.5.1 software package (BioNano Genomics, San Diego, CA) was used to map the assembly scaffolds to the optical map contigs. After a manual curation step where false joins and misassembled contigs were detected by inspection of the alignment, IrysView scaffolding reduced the number of scaffolds to 50 while the scaffold L50 decreased to 12 and the scaffold N50 increased to 108 Mb. Finally, approximately 54k SNP markers from the bovine recombination map[12] were used to detect mis-assemblies and scaffold the autosomes[13]. Markers were aligned to the optical map scaffolds with BLAST[14] requiring 98% mapping identity over the full marker sequence length. Only unique mapping SNPs were considered. Scaffolds were broken when two or more markers from different linkage groups aligned to them. Pearson correlation coefficients between scaffold marker alignment order and genetic map marker order were used to calculate the most probable scaffold order and orientation. Another round of polishing was undertaken with Arrow with the SMRT Analysis 3.1.1 software package.

Gap filling was first done by aligning two Canu v1.4[15] assemblies (run with different overlap algorithms implemented within Canu for error correction, MHAP[16] and minimap[17]) to the scaffolded assembly and identifying alignments crossing gaps. A gap was filled if either assembly spanned a gap with >5000 bp aligning on either side of the gap up to at most 10 bp away from the gap. In the case of a negative gap (i.e. the assemblies had a collapse), both assemblies had to agree on the position and size of the collapse. In total, 171 gaps were closed with this approach. Finally, PBJelly (pbsuite v.15.8.24)[18] was used to fill an additional 91 gaps. The closing of gaps between contigs increased the contig N50 from 12 Mb to 21 Mb and reduced the number of gaps in the genome to 459.

c) Manual curation

Following gap filling, the X chromosome was manually curated using two assemblies produced from MaSuRCA[19] error corrected reads (PacBio corrected with Illumina). The first used Canu v1.4 to assemble the MaSuRCA corrected reads and the other used Celera Assembler[16] version 8.3. MUMmer 3.0[20] alignments between these two assemblies and the gap filled assembly were used to confirm or revise the order and orientation of X chromosome contigs as well as place additional unplaced contigs and scaffolds.

The resulting assembly structure was then re-assessed with an independent genetic map UMCLK (Table S2, Supplementary Note). The BLAT alignment tool[21] and BWA MEM[22] were used to map the probe and flanking sequences present on commercially available genotyping assays to

identify misassemblies. Assembly gaps, Illumina read depth coverage and alignments with dbSNP sequences and flanking sequences were used to refine breakpoints for sequence rearrangements using a combination of custom scripts in iterative fashion[23]. In all, corrections were made to chromosomes 1, 2, 5-12, 16, 18-21, 23, 26, 27, and X. PBJelly was run on the curated assembly to close remaining gaps. The number of gaps decreased from 459 to 386 indicating that our manual curation correctly oriented contigs such that PBJelly could now fill an additional 73 gaps that could not previously be filled. The remaining gaps represent regions where either the gap is too large for our PacBio reads to span, read coverage is low or missing, or there is a remaining misassembly. The contig N50 also increased again from 21 Mb to 26 Mb. Polishing of the assembly proceeded through one iteration of Arrow with all the raw PacBio reads followed by polishing with short Illumina reads (SRR2226514 and SRR2226524 as well as LIB24773 and one run, 97.6 Gb, of LIB18483) using Pilon v1.22[24] with the parameters "--diploid --fix indels –nostrays". The final version of the genome (ARS-UCD1.2) contains 2,628,394,923 bp on the 30 chromosomes (Figure 1b) with an additional 87.5 Mb of unplaced sequence and is available from NCBI under the accession GCF_002263795.1.

d) RNA sequencing

The Iso-Seq method for sequencing full-length transcripts was developed by Pacific Biosciences during the same time period as the genome assembly. We therefore employed this technique to improve characterization of transcript isoforms expressed in cattle tissues, using a diverse set of tissues collected from L1 Dominette 01449 upon euthanasia. The data was collected using an early version of the Iso-Seq library protocol[25] as suggested by Pacific Biosciences. Briefly,

RNA was extracted from each tissue using Trizol reagent as directed (Thermofisher). Two micrograms of RNA was then selected for PolyA tails, and converted into cDNA using the SMARTer PCR cDNA Synthesis Kit (Clontech). The cDNA was amplified in bulk with 12-14 rounds of PCR in eight separate reactions, then pooled and size selected into 1-2 kb, 2-3 kb, and 3-6 kb fractions using the BluePippin instrument (Sage Science). Each size fraction was separately re-amplified in eight additional reactions of 11 PCR cycles. The products for each size fraction amplification were pooled and purified using AMPure PB beads (Pacific Biosciences) as directed, and converted to SMRTbell libraries using the Template Prep Kit v1.0 (Pacific Biosciences) as directed. Iso-Seq was conducted for 22 tissues including abomasum, aorta, atrium, cerebral cortex, duodenum, hypothalamus, jejunum, liver, longissimus dorsi muscle, lung, lymph node, mammary gland, medulla oblongata, omasum, reticulum, rumen, subcutaneous fat, temporal cortex, thalamus, uterine myometrium, and ventricle from the reference cow as well as the testis of her sire. The size fractions were sequenced in either four (for the smaller two fractions) or five (for the largest fraction) SMRTcells on the RSII instrument. Isoforms were identified using the Cupcake ToFU pipeline[26] without using a reference genome.

Due to library size selection and loading bias, Iso-Seq is not reliable for quantitative measurements of transcript abundance. Therefore, we used a combination of public datasets and newly sequenced tissues to annotate the assembly. The GenBank database includes a number of short read-based datasets derived from tissues of Dominette (Table S1), as her tissues have been a freely-distributed resource for the research community. All public data was used in annotation, as well as additional data generated specifically to enhance the assembly while avoiding overlap

with existing public data. Specifically, the TruSeq stranded mRNA LT kit (Illumina, Inc) was used as directed to create RNAseq libraries, which were sequenced to a minimum of 30 million reads for each tissue sample. The Dominette tissues that were sequenced in this study include abomasum, anterior pituitary, aorta, atrium, bone marrow, cerebellum, duodenum, frontal cortex, hypothalamus, KPH fat, lung, lymph node, mammary gland (lactating), medulla oblongata, nasal mucosa, omasum, reticulum, rumen, subcutaneous fat, temporal cortex, thalamus, uterine myometrium, and ventricle. RNAseq libraries were also sequenced from the testis of her sire.

e**) Annotation**

The NCBI Eukaryotic Genome Annotation Pipeline was used to annotate genes, transcripts, proteins and other genomic features on ARS-UCD1.2. Nearly 13 billion RNAseq reads from over 50 tissues and 553,798 consensus Iso-Seq reads from 23 tissues were retrieved from SRA (Table S1) and aligned to the masked genome, along with 12,472 known RefSeq transcripts, 19,820 GenBank transcripts, and 1,583,270 ESTs, using BLAST[14] followed by Splign[27]. The set of proteins aligned to the masked genome consisted of 13,381 RefSeq proteins and 16,371 GenBank proteins from cattle, and 50,089 RefSeq proteins from human. The gene models' structures and boundaries were primarily derived from these alignments. Where alignments did not define a complete model but the coding propensity of the region was sufficiently high, *ab initio* extension or joining/filling of partial ORFs in compatible frame was performed by Gnomon[28], using a hidden Markov model trained on cattle. tRNAs were predicted with tRNAscan-SE:1.23[29] and small non-coding RNAs were predicted by searching the RFAM 12.0 HMMs for eukaryotes using cmsearch from the Infernal package[30]. The annotation of the ARS-UCD1.2 assembly, Annotation Release 106 (AR 106[31]) resulted in 21,039 protein-coding genes, 9,357 non-coding genes and 4,569 pseudogenes.

Data Validation and quality control

Quality assessment

To assess the error profile of our assembly and compare it to the previous reference, UMD3.1.1, (NCBI accession GCF_000003055.5) long- and short-read sequences from Dominette were aligned to both assemblies. Short-read BWA alignments of LIB18483 sequences not used for polishing were evaluated from feature response curves computed with FRCbam[32] (Figure 2a). The total number of erroneous features in ARS-UCD1.2 decreased by over 20% compared to UMD3.1.1 (Table 1). Errors on the chromosome scaffolds exhibited a > 40% reduction in error features compared to UMD3.1.1, suggesting that ARS-UCD1.2 chromosomes were better representative of the individual sequenced. The error classes most prevalent on the ARS-UCD1.2 unplaced sequences compared to the chromosomes were HIGH COV PE, HIGH NORM COV PE, and HIGH SPAN PE with unplaced sequences accounting for 73%, 80%, and 65% of the errors in each class respectively. The increased percentage of HIGH COV PE and HIGH NORM COV PE errors indicates that many of the unplaced sequences are over-assembled or collapsed while HIGH SPAN PE errors would be expected as the majority of the 2181 unplaced sequences are shorter than 25 kb. The same short-read alignments were also used to estimate the quality value (QV) of the assembly with ARS-UCD1.2 scoring 48.67 and UMD3.1.1 37.98, which correspond to a per-base error rate of 1.58×10^{-5} and 1.59×10^{-4}, respectively, or an order-of-magnitude improvement in accuracy. This was calculated from the number of non-matching base calls from FreeBayes[33] as previously described[6]. UMD3.1.1’s lower per-base accuracy resulted from the large number of gaps in the assembly, the larger proportion of unplaced contigs

and the incomplete resolution of larger repetitive regions. In order to further assess the structural integrity of both assemblies, we used Sniffles[34] to evaluate the concordance of long reads from Dominette on both assemblies. All SV classes showed sharp declines in prevalence in ARS-UCD1.2 vs UMD3.1.1 (Table 1). Deletions, duplications, insertions, and inversions all declined by at least 98%.

Table 1. Assembly quality score value statistics, calculations for whole assembly and chromosomes only.

	ARS-UCD1.2	UMD3.1.1	Description
QV	48.67	37.98	Quality value estimate (Phred-scale)
FRCbam output			
Total Features	177889, 128975	230462, 223534	All erroneous features
COMPR PE	37309, 30643	54602, 52606	Areas with low Compression/Expansion statistics
STRECH PE	37255, 22741	35766, 35299	Areas with high CE statistics
HIGH COV PE	7166, 1970	7711, 6331	High read coverage areas (all aligned reads)
HIGH NORM COV PE	5641, 1125	7109, 5778	High paired-read coverage areas (only properly aligned pairs)
HIGH OUTIE PE	139, 102	2108, 2108	Regions with high numbers of misoriented or distant pairs
HIGH SINGLE PE	60, 53	1258, 1256	Regions with high numbers of unmapped pairs
HIGH SPAN PE	4882, 1687	4172, 3582	Regions with high numbers of pairs that map to different scaffolds
LOW COV PE	43370, 36062	57176, 56648	Low read coverage areas (all aligned reads)
LOW NORM COV PE	42067, 34592	60560, 59926	Low paired-end coverage areas (only properly aligned pairs)
Sniffles Output			
DEL	188	10504	Deletions
DUP	16	728	Duplications
INS	106	4911	Insertions
INV	34	2675	Inversions

Improved contiguity

A key measure of improvement over the previous reference is the increase in the contiguity of the genome (Figure 1). The 30 cattle chromosomes are now composed of 345 contigs compared to 72,264 contigs in the UMD3.1.1 assembly. This represents a 280-fold increase in the contig NG50 (N50 calculated from a fixed 2.8Gb genome size), from 0.092 Mb to 25.8 Mb (Figure 2b) and a 209-fold increase in sequence continuity. The 345 contigs in ARS-UCD1.2 equate to 315 gaps in the chromosomes vs. 72,234 on UMD3.1.1. We demonstrated the impact of higher contiguity on the mapping of existing datasets by aligning the currently-available 14,473 known cattle RefSeq transcripts (with accession prefixed with NM_ and NR_) to both ARS-UCD1.2 and. We found that the transcripts aligned more cleanly to ARS-UCD1.2 than to UMD3.1.1 (Table 2). The number of transcripts for which the best alignment covered less than 95% of the CDS went down from 734 on UMD3.1.1 to only 37 for ARS-UCD1.2. Moreover, the alignment of 219 transcripts were split across two or more genomic sequences of UMD3.1.1 compared to only 9 for ARS-UCD1.2.

Table 2: Splign alignment of RefSeq transcripts to ARS-UCD1.2 and UMD3.1.1

Name	ARS-UCD1.2	UMD3.1.1
Accession	GCF_002263795.1	GCF_000003055.5
Number of sequences retrieved from Entrez	14,473	14,473
Number of sequences not aligning	19	13
Number of sequences with multiple best alignments (split genes)	9	219
Number of sequences with CDS coverage < 95%	37	734

Annotation comparison

The ARS-UCD1.2 assembly annotation (AR 106) generated by NCBI was compared to the UMD3.1.1 annotation (NCBI Bos taurus Annotation Release 105, AR 105[35]). About 2/3 of the genes (85% of protein-coding genes) are identical or nearly identical between the two datasets. Over 90% of the novel genes (19% of total genes) in AR 106 were non-coding genes, due in part to the addition of a module for the prediction of short non-coding genes based on RFAM models to the annotation pipeline after AR 105 was produced. The number of protein-coding genes with at least one isoform covering 95% of the length of a UniProt/SwissProtKB protein is 17,810 (85% of protein-coding genes) for AR 106 versus 16,956 (80%) for AR 105, suggesting that the protein models predicted in AR 106 are generally more complete than in AR 105.

These improvements in the annotation are partly due to the availability of more and longer transcript evidence for gene prediction (Iso-Seq in particular), but it is clear that uncertainty of placement and orientation of sequence across gaps has a large impact on gene annotation. Of the 21,039 genes annotated in ARS-UCD1.2, 69 (0.3%) have gaps within introns compared to 6949 (33%) of annotated UMD3.1.1 genes (Figure 2c). Considering the potential impact of regulatory elements flanking genes, it is also important to note that almost 60% of UMD3.1.1 genes have gaps within 10 kb while that percentage drops below 1% in ARS-UCD1.2.

ARS-UCD1.2 also represents an improvement in base accuracy over UMD3.1.1 that is measurable in the annotation. High rates of sequencing error can disrupt the prediction of open reading frames and lead to truncated gene models or the erroneous calling of non-coding genes or pseudogenes instead of protein-coding genes. The NCBI annotation process attempts to compensate for this problem by producing a ‘corrected’ model (with name prefixed with LOW QUALITY) containing a difference with the genome sequence, when protein alignments suggest

there is an erroneous indel in the genome. The number of such ‘corrected’ models decreased by 44% from 1,828 in UMD3.1.1/AR 105 to 1,027 in ARS-UCD1.2/AR 106,

Conclusions

This assembly represents a 200-fold improvement in sequence continuity and a 10-fold improvement in per-base accuracy over previous cattle assemblies. The assignment of megabase-length contigs to full chromosome scaffolds provides additional certainty in gene and genetic marker positions which will influence marker-assisted selection and basic research. The assembly was selected as the reference genome for taurine cattle by the US genomic evaluation system in December 2018[36] and the 37 partner institutions of the 1000 Bull Genomes Project for the run7 variant calls distributed globally in June 2019[37]. We demonstrate that assembly improvements warranted adoption by these projects and that increased assembly accuracy will benefit future genetics research on this species.

Availability of supporting data and materials

Accession numbers for raw sequencing reads and assemblies can be found in Table S1.

Additional files

Table S1. Sequencing resources

Table S2. UMCLK genetic map

Supplemental Note. UMCLK genetic map

Abbreviations

bp: base pairs; BWA: Burrows-Wheeler Aligner; Gb: gigabase pairs; HMW: high molecular weight; kb: kilobase pairs; Mb: megabase pairs; NCBI: National Center for Biotechnology Information; RefSeq: Reference Sequence; RNAseq: high-throughput short-read messenger RNA sequencing; SMRT: single molecule real-time; SNP: single-nucleotide polymorphism; SRA: Sequence Read Archive; SV: structural variant; tRNA: transfer RNA

Competing interests

RH and ET are employed by Pacific Biosciences, all other authors declare that they have no competing interests.

Author contributions

TPLS, JFM, CPVT, RDS, DMB, and BDR conceived, initiated, and managed the project. TPLS and JFM were responsible for DNA and RNA sequence data production. BDR, DMB, RDS, SJS, CD, AZ, RH, JG, AR, SK, and AMP performed assembly and associated tasks. TNR, WYL, CC, WL, SDM, BMM, WMS, JAH, JCS, WN, SGS, JBC, GL, and CPVT performed quality control and/or contributed additional analyses. CGE, TGM, and ET performed RNA analyses. FTN and JH performed annotation and managed public presentation of the assembly files. All authors read and edited the manuscript.

Acknowledgements

Sequence generation was supported by USDA/NRSP-8 Animal Genome, USDA-ARS Meat Animal Research Center, Neogen and Zoetis. BDR, SGS, JBC, CPVT, and GL were supported by USDA CRIS 8042-31000-001-00-D. JBC was supported by USDA CRIS 8042-31000-002-00-D. DMB and WL were supported in part by USDA CRIS 5090-31000-026-00-D. DMB was also supported in part by USDA NIFA grant 5090-31000-026-06-I. WMS, TGM, TPLS were supported by USDA CRIS 3040-31000-100-00-D. RDS and TNR were supported in part by

USDA NIFA 2016-68004-24827. RDS was also supported in part by USDA NIFA 2013-67015-21202, 2015-67015-23183, NIH 1R01HD084353-01A1 and USDA Hatch MO-HAAS0001. JH and FTN were supported by the Intramural Research Program of the National Library of Medicine, National Institutes of Health. JAH and JCS were supported by funding from the UKRI-BBSRC awards BB/M027155/1, BBS/E/I/00007035, BBS/E/I/00007038 and BBS/E/I/00007039. SK, AR, and AMP were supported by the Intramural Research Program of the National Human Genome Research Institute, National Institutes of Health. This work utilized the computational resources of the NIH HPC Biowulf cluster (https://hpc.nih.gov).

References

1. Robinson, T. P. *et al.* Mapping the Global Distribution of Livestock. *PLOS ONE* **9**, e96084 (2014).
2. Weigel, K. A., VanRaden, P. M., Norman, H. D. & Grosu, H. A 100-Year Review: Methods and impact of genetic selection in dairy cattle—From daughter–dam comparisons to deep learning algorithms. *J. Dairy Sci.* **100**, 10234–10250 (2017).
3. Saatchi, M., Schnabel, R. D., Rolf, M. M., Taylor, J. F. & Garrick, D. J. Accuracy of direct genomic breeding values for nationally evaluated traits in US Limousin and Simmental beef cattle. *Genet. Sel. Evol.* **44**, 38 (2012).
4. García-Ruiz, A. *et al.* Changes in genetic selection differentials and generation intervals in US Holstein dairy cattle as a result of genomic selection. *Proc. Natl. Acad. Sci. U. S. A.* **113**, E3995–E4004 (2016).
5. Consortium, T. B. G. S. and A., Elsik, C. G., Tellam, R. L. & Worley, K. C. The Genome Sequence of Taurine Cattle: A Window to Ruminant Biology and Evolution. *Science* **324**, 522–528 (2009).

6. Bickhart, D. M. *et al.* Single-molecule sequencing and chromatin conformation capture enable de novo reference assembly of the domestic goat genome. *Nat. Genet.* **49**, 643–650 (2017).
7. Chin, C.-S. *et al.* Phased diploid genome assembly with single-molecule real-time sequencing. *Nat. Methods* **13**, 1050–1054 (2016).
8. Chin, C.-S. *et al.* Nonhybrid, finished microbial genome assemblies from long-read SMRT sequencing data. *Nat. Methods* **10**, 563–569 (2013).
9. Chaisson, M. J. & Tesler, G. Mapping single molecule sequencing reads using basic local alignment with successive refinement (BLASR): application and theory. *BMC Bioinformatics* **13**, 238 (2012).
10. Putnam, N. H. *et al.* Chromosome-scale shotgun assembly using an in vitro method for long-range linkage. *Genome Res.* **26**, 342–350 (2016).
11. Zhou, S. *et al.* A clone-free, single molecule map of the domestic cow (Bos taurus) genome. *BMC Genomics* **16**, 644 (2015).
12. Ma, L. *et al.* Cattle Sex-Specific Recombination and Genetic Control from a Large Pedigree Analysis. *PLOS Genet.* **11**, e1005387 (2015).
13. KHP-Informatics/illumina-array-protocols. *GitHub* Available at: https://github.com/KHP-Informatics/illumina-array-protocols. (Accessed: 6th September 2019)
14. Altschul, S. F., Gish, W., Miller, W., Myers, E. W. & Lipman, D. J. Basic local alignment search tool. *J. Mol. Biol.* **215**, 403–410 (1990).
15. Koren, S. *et al.* Canu: scalable and accurate long-read assembly via adaptive k-mer weighting and repeat separation. *Genome Res.* gr.215087.116 (2017). doi:10.1101/gr.215087.116

16. Berlin, K. *et al.* Assembling large genomes with single-molecule sequencing and locality-sensitive hashing. *Nat. Biotechnol.* **33**, 623–630 (2015).

17. Li, H. Minimap and miniasm: fast mapping and de novo assembly for noisy long sequences. *Bioinformatics* **32**, 2103–2110 (2016).

18. English, A. C. *et al.* Mind the Gap: Upgrading Genomes with Pacific Biosciences RS Long-Read Sequencing Technology. *PLOS ONE* **7**, e47768 (2012).

19. Zimin, A. V. *et al.* Hybrid assembly of the large and highly repetitive genome of Aegilops tauschii, a progenitor of bread wheat, with the MaSuRCA mega-reads algorithm. *Genome Res.* **27**, 787–792 (2017).

20. Kurtz, S. *et al.* Versatile and open software for comparing large genomes. *Genome Biol.* **5**, R12 (2004).

21. Kent, W. J. BLAT—The BLAST-Like Alignment Tool. *Genome Res.* **12**, 656–664 (2002).

22. Li, H. Aligning sequence reads, clone sequences and assembly contigs with BWA-MEM. *ArXiv13033997 Q-Bio* (2013).

23. Bickhart, D. *Scripts and documentation related to the assembly of ARS-UCD1.2: njdbickhart/CattleAssemblyScripts*. (2019).

24. Walker, B. J. *et al.* Pilon: An Integrated Tool for Comprehensive Microbial Variant Detection and Genome Assembly Improvement. *PLOS ONE* **9**, e112963 (2014).

25. Procedure & Checklist - Isoform Sequencing (Iso-Seq^{TM}) using the Clontech SMARTer PCR cDNA Synthesis Kit and Manual Agarose-gel. 16

26. Tseng, E. *Miscellaneous collection of Python and R scripts for processing Iso-Seq data: Magdoll/cDNA_Cupcake*. (2019).

27. Kapustin, Y., Souvorov, A., Tatusova, T. & Lipman, D. Splign: algorithms for computing spliced alignments with identification of paralogs. *Biol. Direct* **3**, 20 (2008).
28. Gnomon - the NCBI eukaryotic gene prediction tool. Available at: https://www.ncbi.nlm.nih.gov/genome/annotation_euk/gnomon/. (Accessed: 8th August 2019)
29. Lowe, T. M. & Eddy, S. R. tRNAscan-SE: A Program for Improved Detection of Transfer RNA Genes in Genomic Sequence. *Nucleic Acids Res.* **25**, 955–964 (1997).
30. Nawrocki, E. P. *et al.* Rfam 12.0: updates to the RNA families database. *Nucleic Acids Res.* **43**, D130–D137 (2015).
31. Bos taurus Annotation Report 106. Available at: https://www.ncbi.nlm.nih.gov/genome/annotation_euk/Bos_taurus/106/. (Accessed: 15th August 2019)
32. Vezzi, F., Narzisi, G. & Mishra, B. Reevaluating Assembly Evaluations with Feature Response Curves: GAGE and Assemblathons. *PLOS ONE* **7**, e52210 (2012).
33. Garrison, E. & Marth, G. Haplotype-based variant detection from short-read sequencing. *ArXiv12073907 Q-Bio* (2012).
34. Sedlazeck, F. J. *et al.* Accurate detection of complex structural variations using single-molecule sequencing. *Nat. Methods* **15**, 461 (2018).
35. Bos taurus Annotation Report 105. Available at: https://www.ncbi.nlm.nih.gov/genome/annotation_euk/Bos_taurus/105/. (Accessed: 15th August 2019)
36. Null, D. J., VanRaden, P. M., Rosen, B. D., O'Connell, J. R. & Bickhart, D. M. Using the ARS-UCD1.2 reference genome in U.S. evaluations. *Interbull Bull.*

37. 1000 Bull Genomes Project. Available at: http://www.1000bullgenomes.com/. (Accessed: 6th September 2019)

Figures

Figure 1. Dominette* de novo *assembly. *A) Assembly pipeline. N50 is the minimum scaffold/contig length needed to cover 50% of the genome. L50 is the number of contigs required to reach N50. B) Cattle chromosomes painted with assembled contigs. A color shift indicates the switch from one contig to the next or the end of an alignment block. The left half of each chromosome shows UMD3.1.1 contigs while the right shows ARS-UCD1.2. To be conservative, contigs were ordered by UMD3.1.1 assembly positions, where there are conflicts in order between ARS-UCD1.2 and UMD3.1.1, the plot will display a color switch in ARS-UCD1.2. *Within scaffolds assigned to chromosomes*

Figure 2. Assembly assessments computed for ARS-UCD1.2 and UMD3.1.1. *A) Feature response curves computed for ARS-UCD1.2 and UMD3.1.1. B) Calculated NG showing a 280-fold increase of ARS-UCD1.2 in comparison to UMD3.1.1. C) The percentage of gaps in gene flaking regions are reduced from 33% to 0.3% in ARS-UCD1.2 in comparison to UMD3.1.1.*

Figure1

Click here to download Figure Figure1.bmp

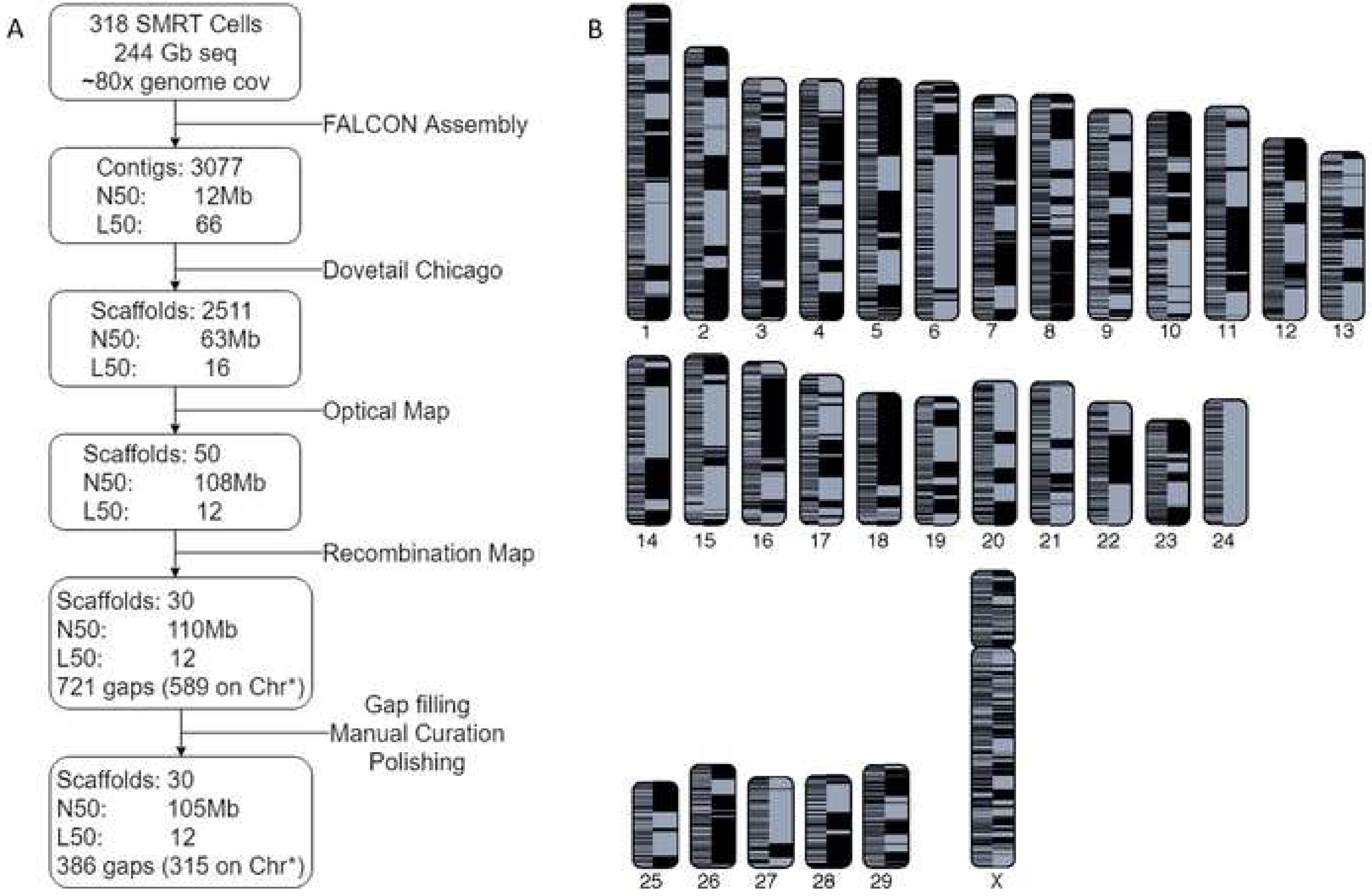

Figure2

Click here to download Figure Figure2.bmp

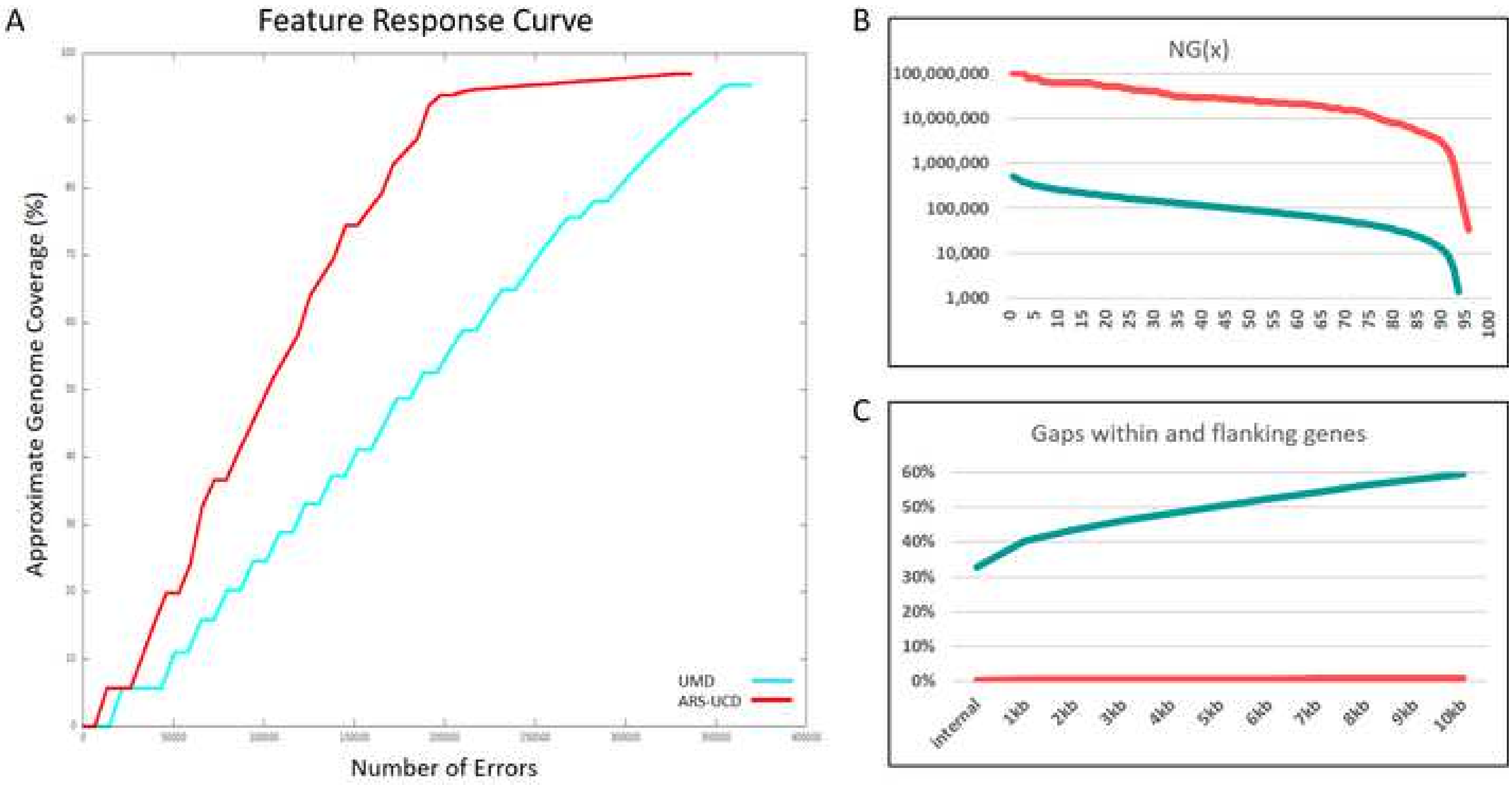

supplementary table

Click here to access/download
Supplementary Material
TableS1_sequencing_resources.xlsx

Click here to access/download
Supplementary Material
SupplementalNote_UMCLK_genetic_map.docx

supplementary table

Click here to access/download

Supplementary Material

TableS2_UMCLK genetic map.csv
