# Supplementary material for: De novo assembly of the cattle reference genome with single-molecule sequencing
Source: Gigascience. 2020 Mar 19;9(3):giaa021. doi: 10.1093/gigascience/giaa021 (PMC7081964; doi:10.1093/gigascience/giaa021)
Supplement: giaa021_GIGA-D-19-00331_Revision_1 [file giaa021_giga-d-19-00331_revision_1.pdf]

GigaScience

De novo assembly of the cattle reference genome with single-molecule sequencing

--Manuscript Draft--

**Manuscript Number:**	GIGA-D-19-00331R1
**Full Title:**	De novo assembly of the cattle reference genome with single-molecule sequencing
**Article Type:**	Data Note

Funding Information:

USDA/NRSP8 Animal Genome	Not applicable
USDA ARS Meat Animal Research Center	Not applicable
Neogen	Not applicable
Zoetis	Not applicable
Agricultural Research Service (8042-31000-001-00-D)	Not applicable
Agricultural Research Service (8042-31000-002-00-D)	Not applicable
Agricultural Research Service (5090-31000-026-00-D)	Not applicable
National Institute of Food and Agriculture (5090-31000-026-06-I)	Dr Derek M Bickhart
Agricultural Research Service (3040-31000-100-00-D)	Not applicable
National Institute of Food and Agriculture (2016-68004-24827,2013-67015-21202, 2015-67015-23183)	Dr Robert D Schnabel
National Institutes of Health (1R01HD084353-01A1)	Dr Robert D Schnabel
USDA Hatch (MO-HAAS0001)	Dr Robert D Schnabel
U.S. National Library of Medicine	Not applicable
Biotechnology and Biological Sciences Research Council (BB/M027155/1, BBS/E/I/00007035, BBS/E/I/00007038 and BBS/E/I/00007039)	Dr John A Hammond
National Human Genome Research Institute	Not applicable

Abstract:

Major advances in selection progress for cattle have been made following the introduction of genomic tools over the past 10-12 years. These tools depend upon the Bos taurus reference genome (UMD3.1.1), which was created using now-outdated technologies and suffers from a variety of deficiencies and inaccuracies. We present the new reference genome for cattle, ARS-UCD1.2, based on the same animal as the original to facilitate transfer and interpretation of results obtained from the earlier version, but applying a combination of modern technologies in a de novo assembly to increase continuity, accuracy, and completeness. The assembly includes 2.7 Gb, and is >250x more continuous than the original assembly, with contig N50 >25 Mb and L50 of 32. We also greatly expanded supporting RNA-based data for annotation that identifies 30,396 total genes (21,039 protein coding). The new reference assembly is accessible in annotated form for public use.

**Corresponding Author:**	Benjamin D Rosen<br><br>UNITED STATES
**Corresponding Author Secondary Information:**	
**Corresponding Author's Institution:**	
**Corresponding Author's Secondary**	

Powered by Editorial Manager® and ProduXion Manager® from Aries Systems Corporation

**Institution:**	
**First Author:**	Benjamin D Rosen
**First Author Secondary Information:**	
**Order of Authors:**	Benjamin D Rosen
	Derek M Bickhart
	Robert D Schnabel
	Sergey Koren
	Christine G Elsik
	Elizabeth Tseng
	Troy N Rowan
	Wai Y Low
	Aleksey Zimin
	Christine Couldrey
	Richard Hall
	Wenli Li
	Arang Rhie
	Jay Ghurye
	Stephanie D McKay
	Francoise Thibaud-Nissen
	Jinna Hoffman
	Brenda M Murdoch
	Warren M Snelling
	Tara G McDaneld
	John A Hammond
	John C Schwartz
	Wilson Nandolo
	Darren E Hagen
	Christian Dreischer
	Sebastian J Schultheiss
	Steven G Schroeder
	Adam M Phillippy
	John B Cole
	Curtis P Van Tassell
	George Liu
	Timothy P.L. Smith
	Juan F Medrano
**Order of Authors Secondary Information:**	
**Response to Reviewers:**	Responses are also included in the Personal Cover with better formatting.<br><br>Reviewer #1:

Powered by Editorial Manager® and ProduXion Manager® from Aries Systems Corporation

Specific comments for revision:
1.It is not clear from section (e) of the Methods how the alignments with UniProt/SwissProtKB were generated (i.e. through BLAST, Splign, or another tool).
REPLY: Text added to the Methods section (e).
"The respective quality of the UMD3.1.1 annotation (Annotation Release 105 AR 105[33]) and AR 106 was evaluated by aligning the annotated proteins of each release to the UniProtKB/SwissProt proteins available in Entrez Protein (returned by the Entrez query srcdb_swiss_prot[properties] AND eukaryotes[orgn] on 7/29/2019) using BlastP. For each protein coding gene, the protein isoform with the best alignment based on score (or in case of a tie, based on alignment length, percent coverage or subject protein length) was chosen as the isoform representative of the gene. The counts of protein coding genes in AR 105 and AR 106 with representative isoforms covering at least 95% of the length of the UniProtKB/SwissProt proteins were then compared."

2.Related to this analysis, which release of UniProt/SwissProtKB was used?
REPLY: Date of access added to new text in Methods section (e).
"(returned by the Entrez query srcdb_swiss_prot[properties] AND eukaryotes[orgn] on 7/29/2019)"

3.Are protein sequences in the UniProt/SwissProtKB data set potentially derived in part from AR 105 or AR 106? Does this complicate interpretation of these results?
REPLY: There is a possibility that some of the proteins in the UniProt/SwissProtKB data set are from AR 105 or AR 106. However, Refseq sequences are not submitted to the International Nucleotide Sequence Database Collaboration and according to the UniProt documentation (https://www.uniprot.org/help/sequence_origin) “More than 95% of the protein sequences provided by UniProtKB come from the translations of coding sequences (CDS) submitted to the EMBL-Bank/GenBank/DDBJ nucleotide sequence resources (International Nucleotide Sequence Database Collaboration”, so the chances are small. The RefSeq proteins that would be incorporated into UniProt/SwissProtKB would have been manually curated and presumably have very strong support from experimental evidence. Whether or not RefSeq proteins are part of the UniProt/SwissProtKB, the strength of the analysis relies in the difference in the number of good hits between the two annotation releases rather than in the absolute numbers of hits for each release.

4.In the 'Annotation comparison' section, the authors state that "About 2/3 of the genes (85% of protein-coding genes) are identical or nearly identical between the two datasets." What qualifies as nearly identical?
REPLY: Nearly identical genes are highly similar genes, with support scores of 0.66 or more (on a scale of 0 to 1) on both sides of the comparison. The support score is derived from a combination of matching exon boundaries and sequence overlap.
Table S5 containing the comparison data was added as well as the following text.
"(with a support score, derived from a combination of matching exon boundaries and sequence overlap, of 0.66 or more, on a scale of 0 to 1, on both sides of the comparison)"

5.Based on information in Table 2, there are six sequences that align to the UMD3.1.1 assembly, but not ARS-UCD1.2. Are these six cases thought to represent bona fide deficiencies in the ARS-UCD1.2 assembly?
REPLY: It’s a bit more complicated than this. The net difference in the count of sequences that do not align is 6, but there are only two transcripts that align to neither assembly. Additionally, the difference is made up by Y-linked transcripts which shouldn’t be found in either assembly. I’ve added this to the text.
"Although a greater number of transcripts failed to align to ARS-UCD1.2, this difference is made up of transcripts from Y-linked genes (Table S4). The presence of Y-linked genes in the UMD3.1.1 assembly is likely due to Y chromosome contamination from the inclusion of sequence from a bacterial artificial chromosome library prepared from Dominette’s sire [34,39]. Since ARS-UCD1.2 is derived from an XX female and does not contain the Y chromosome, we recommend the inclusion of an independently assembled Y chromosome prior to analysis as is being done by the 1000 Bull Genomes Project [40]."

6.In the "Improved contiguity" section, I suggest explaining to the reader the relevance

Powered by Editorial Manager® and ProduXion Manager® from Aries Systems Corporation

	of "accession prefixed with NM_ and NR_".<br>REPLY: Added “a manually curated set of transcript accessions” to clarify.<br>"(a manually curated set of transcript accessions prefixed with NM_ and NR_)"<br><br>7.In Table 2 the label "Number of sequences with multiple best alignments (split genes)" could be improved, as the meaning of "multiple best alignments" isn't obvious in this context.<br>REPLY: Changed.<br>"Number of sequences whose best alignments span multiple loci (split genes)"<br><br>8.Change last comma to period in "1,027 in ARS-UCD1.2/AR 106,"<br>REPLY: Fixed<br><br>9.Fix truncated sentence "to both ARS-UCD1.2 and."<br>REPLY: Fixed<br>"to both ARS-UCD1.2 and UMD3.1.1."<br><br>10.It isn't clear how citations 23 and 26 will be useful, at least in their current form. Perhaps in the published article they will link to the corresponding scripts.<br>REPLY: Proper URLs inserted, references are now 24 and 27.<br>"24.Bickhart, D. Scripts and documentation related to the assembly of ARS-UCD1.2: https://github.com/njdbickhart/CattleAssemblyScripts. (2019)."<br>"27.Tseng, E. Miscellaneous collection of Python and R scripts for processing Iso-Seq data: https://github.com/Magdoll/cDNA_Cupcake. (2019)."<br><br>11.Regarding the UMCLK genetic map supplementary file, is the provided SQL to be used with Crimap?<br>REPLY: The provided SQL was used to generate the TableS2 UMCLK genetic map.csv file which is included with the manuscript, text has been added to the supplementary note to clarify.<br>"The linkage map is stored in a PostgreSQL database at the University of Missouri. The SQL below was used to generate the TableS2_UMCLK genetic map.csv file which is included with the manuscript."<br>"The following is a description of file TableS2_UMCLK genetic map.csv which is comma delimited with a header row. Fields are specified as below including a description of the field."<br><br>Reviewer #2:<br><br>The manuscript could be improved by addressing the following issues:<br>1. Was the Dovetail Chicago library constructed from DNA/chromatin from the same individual as the genome sequence data? If so, then it would be useful to confirm this. If not, then it would be useful to comment on whether this limited the accuracy of the scaffolding.<br>REPLY: Yes, added “from Dominette lung tissue” to clarify.<br>"First, a Chicago library was prepared as described previously[10] from Dominette lung tissue"<br><br>2. Similarly, was the optical map generated from DNA from the same individual as the genome sequence data?<br>REPLY: Yes, added “Dominette derived” for clarification.<br>"Next we used the Dominette derived Bos taurus optical map BtOM1.0"<br><br>3. In terms of the completeness of the assembly, did the authors detect centromeric and telomeric sequences in the chromosome assigned scaffolds?<br>REPLY: This is a good question. We have added this information to the “Quality assessment” section including Table S3 and Figure S1.<br>"As a measure of the completeness of the assemblies and to define the chromosome ends, we identified centromeric[24] and telomeric[37] repeats (Table S3). For the 29 acrocentric autosomes, we identified the expected centromeric and telomeric repeats on 9 ARS-UCD1.2 chromosomes (5,6,8,10,13,14,16,17, and 18) whereas no UMD3.1.1 chromosomes contained both, mainly due to a relative lack of telomeric repeats in the assembly. ARS-UCD1.2 chromosomes 3,20, and 22 are missing both chromosome ends, while chromosomes 1,9,10, and 15 erroneously contain

centromeric repeats at both ends. Finally, the metacentric X chromosome only has telomeric repeats at one end and no centromeric repeats. Telomeric repeats were only identified on UMD3.1.1 chromosome 20, centromeric repeats are found on the proper end of 22 autosomes (missing on 6,7,20,21,22,27, and 28) and the X chromosome contains centromeric repeats. All chromosomes also contain centromeric repeats dispersed throughout so it is difficult to determine if the X centromere is properly placed. Centromeric repeat regions at the start of ARS-UCD1.2 chromosome scaffolds were over 2-fold larger than their counterparts in the UMD3.1 reference (Figure S1)."

4. Why was manual curation of the assembly limited to the X chromosome?
REPLY: The X chromosome was just the first to be manually curated, the entire assembly was manually curated “In all, corrections were made to chromosomes 1, 2, 5-12, 16, 18-21, 23, 26, 27, and X”. We have reworded the beginning of the Methods section (c), Manual curation, to clarify.
"Following gap filling, the assembly was manually curated. To start, we assessed the X chromosome using two assemblies produced from MaSuRCA[20] error-corrected reads (PacBio corrected with Illumina). The first used Canu v1.4 to assemble the MaSuRCA corrected reads and the other used Celera Assembler[17] version 8.3. MUMmer 3.0[21] alignments between these two assemblies and the gap-filled assembly were used to confirm or revise the order and orientation of X-chromosome contigs as well as place additional unplaced contigs and scaffolds. Next, the autosomal assembly structure was manually curated and oriented with an independent genetic map UMCLK (Table S2, Supplementary Note). The BLAT alignment tool[22] and BWA MEM[23] were used to map the probe and flanking sequences present on commercially available genotyping assays to identify misassemblies. Assembly gaps, Illumina read-depth coverage and alignments with dbSNP sequences and flanking sequences were used to refine breakpoints for sequence rearrangements using a combination of custom scripts in iterative fashion[24]. In all, corrections were made to chromosomes 1, 2, 5-12, 16, 18-21, 23, 26, 27, and X."

5. The second of these two sentences is a non-sequitur "Due to library size selection and loading bias, Iso-Seq is not reliable for quantitative measurements of transcript abundance. Therefore, we used a combination of public datasets and new sequenced tissues to annotate the assembly." The rationale underpinning use of other expression data (short read RNA-Seq, cDNA and ESTs) for genome annotation was presumably that the Iso-Seq data provided insufficient sequence depth to allow lowly expressed transcripts to be detected. The short read RNA-Seq, cDNA and ESTs data presumably also allowed transcripts that are restricted to other tissues, cell types, developmental stages, states and sex to be captured in the annotation.
REPLY: Agree, reworded.
"Short read based RNA-seq data derived from tissues of Dominette were available in the GenBank database, as her tissues have been a freely-distributed resource for the research community. To complement and extend this data, and to ensure that the tissues used for Iso-Seq were also represented by RNA-seq data for quantitative analysis and confirmation of isoforms observed in Iso-Seq, we generated additional data avoiding overlap with existing public data."

6. What is KPH fat as sampled by the authors? KPH fat appears to be fat from kidney, pelvis and heart. Did the authors sample fat from all three of these depots and then pool them before or after preparing RNA in order to make the relevant sequence library?
REPLY: KPH fat refers to internal organ fat as opposed to subcutaneous fat. Generally speaking, and in this case, the sample is taken from the covering on the kidney capsule.
"(internal organ fat taken from the covering on the kidney capsule)"

7. Table 1 is poorly laid out. From the title of the Table it seems likely that the first number in each column, in which there are two numbers, refers to the whole assembly and the second to the chromosomes only. This needs to be more explicit with a footnote or legend. As the comparisons made in the text refer to the statistics for the chromosomes and the unplaced scaffolds it would be better to present these numbers in the Table rather than the statistics for the whole assembly and the chromosomes, thus requiring the reader to calculate the numbers for the unplaced scaffolds. The appearance of the Table would be improved by dividing the columns with two entries

Powered by Editorial Manager® and ProduXion Manager® from Aries Systems Corporation

	into two columns. The appearance of this and other Tables with numbers would also be improved by right justifying the numbers.<br>REPLY: Table reformatted.<br><br>8. The use of separators for 1,000s and large numbers in the manuscript is inconsistent. These large numbers are much more readable with "," separators.<br>REPLY: Agree, fixed.<br><br>9. What was the basis for assigning and orienting scaffolds to/on chromosomes? The linkage map(s) like the sequence assemblies are agnostic about chromosome assignment and orientation on chromosomes. There is no doubt historical data linking specific genes and sequences to particular chromosome locations from cytogenetic analysis. It would be helpful to make these links explicit.<br>REPLY: The scaffolding section describes the use of the recombination map to scaffold the chromosomes. The UMCLK linkage map was used to orient the chromosomes. “oriented with” has been added to the text.<br>"Finally, approximately 54k SNP markers from the bovine recombination map[12] were used to detect mis-assemblies and scaffold the 29 acrocentric autosomes."<br>"The resulting assembly structure was then re-assessed, manually curated, and oriented with an independent genetic map UMCLK."
**Additional Information:**	
**Question**	**Response**
Are you submitting this manuscript to a special series or article collection?	No
**Experimental design and statistics**<br><br>Full details of the experimental design and statistical methods used should be given in the Methods section, as detailed in our Minimum Standards Reporting Checklist. Information essential to interpreting the data presented should be made available in the figure legends.<br><br>Have you included all the information requested in your manuscript?	Yes
**Resources**<br><br>A description of all resources used, including antibodies, cell lines, animals and software tools, with enough information to allow them to be uniquely identified, should be included in the Methods section. Authors are strongly encouraged to cite Research Resource Identifiers (RRIDs) for antibodies, model organisms and tools, where possible.<br><br>Have you included the information	Yes

requested as detailed in our Minimum Standards Reporting Checklist?	
**Availability of data and materials**<br><br>All datasets and code on which the conclusions of the paper rely must be either included in your submission or deposited in publicly available repositories (where available and ethically appropriate), referencing such data using a unique identifier in the references and in the "Availability of Data and Materials" section of your manuscript.<br><br>Have you have met the above requirement as detailed in our Minimum Standards Reporting Checklist?	Yes

Manuscript

Click here to access/download;Manuscript;ars-ucd-datanote_revision_forpublication.docx

GIGASCIENCE, DATA NOTE

De novo assembly of the cattle reference genome with single-molecule sequencing

Benjamin D. Rosen[1†*] ben.rosen@usda.gov (Corresponding Author), Derek M. Bickhart[2†] derek.bickhart@usda.gov, Robert D. Schnabel[3†] schnabelr@missouri.edu, Sergey Koren[4] sergey.koren@nih.gov, Christine G. Elsik[3] elsikc@missouri.edu, Elizabeth Tseng[5] etseng@pacificbiosciences.com, Troy N. Rowan[3] tnr343@mail.missouri.edu, Wai Y. Low[6] wai.low@adelaide.edu.au, Aleksey Zimin[7,8] alekseyz@jhu.edu, Christine Couldrey[9] christine.couldrey@lic.co.nz, Richard Hall[5] rhall@pacificbiosciences.com, Wenli Li[2] wenli.li@usda.gov, Arang Rhie[4] rhiea@nih.gov, Jay Ghurye[10] jayg@cs.umd.edu, Stephanie D. McKay[11] stephanie.mckay@uvm.edu, Françoise Thibaud-Nissen[12] thibaudf@ncbi.nlm.nih.gov, Jinna Hoffman[12] jinna.choi@nih.gov, Brenda M. Murdoch[13] bmurdoch@uidaho.edu, Warren M. Snelling[14] warren.snelling@usda.gov, Tara G. McDaneld[14] tara.mcdaneld@usda.gov, John A. Hammond[15] john.hammond@pirbright.ac.uk, John C. Schwartz[15] john.schwartz@pirbright.ac.uk, Wilson Nandolo[16,17] wilsonandolo@gmail.com, Darren E. Hagen[18] darren.hagen@okstate.edu Christian Dreischer[19] christian.dreischer@computomics.com, Sebastian J Schultheiss[19] sebastian.schultheiss@computomics.com, Steven G. Schroeder[1] steven.schroeder@usda.gov, Adam M. Phillippy[4] adam.phillippy@nih.gov, John B. Cole[1] john.cole@usda.gov, Curtis P. Van Tassell[1] curt.vantassell@usda.gov, George Liu[1] george.liu@usda.gov, Timothy P.L. Smith[14*] tim.smith2@usda.gov (Corresponding Author), Juan F. Medrano[20] jfmedrano@ucdavis.edu

Affiliations

[1]Animal Genomics and Improvement Laboratory, USDA-ARS, Beltsville, MD, USA

[2]Dairy Forage Research Center, USDA-ARS, Madison, WI, USA

[3]University of Missouri, Columbia, MO, USA

[4]Genome Informatics Section, Computational and Statistical Genomics Branch, National Human Genome Research Institute, National Institutes of Health, Bethesda, MD, USA

[5]Pacific Biosciences, Menlo Park, CA, USA

[6]The Davies Research Centre, University of Adelaide, Roseworthy, Australia

[7]Johns Hopkins School of Medicine, Baltimore, MD, USA

[8]Johns Hopkins University, Baltimore, MD, USA

[9]Livestock Improvement Corporation, Hamilton, New Zealand

[10] University of Maryland, College Park, MD, USA

[11]University of Vermont, Burlington, VT, USA

[12]National Center for Biotechnology Information, National Library of Medicine, National Institutes of Health, Bethesda, MD, USA

[13]University of Idaho, Moscow, ID, USA

[14]U.S. Meat Animal Research Center, USDA-ARS, Clay Center, NE, USA

[15]The Pirbright Institute, Woking, UK

[16]University of Natural Resources and Life Sciences, Vienna, Austria

[17]Lilongwe University of Agriculture and Natural Resources, Lilongwe, Malawi

[18]Oklahoma State University, Stillwater, OK, USA

[19]Computomics GmbH, Tuebingen, Germany

[20]University of California, Davis, CA, USA

[†]These authors contributed equally to this work

*Correspondence: ben.rosen@usda.gov, tim.smith2@usda.gov

Abstract

Major advances in selection progress for cattle have been made following the introduction of genomic tools over the past 10-12 years. These tools depend upon the *Bos taurus* reference genome (UMD3.1.1), which was created using now-outdated technologies and suffers from a variety of deficiencies and inaccuracies.

We present the new reference genome for cattle, ARS-UCD1.2, based on the same animal as the original to facilitate transfer and interpretation of results obtained from the earlier version, but applying a combination of modern technologies in a *de novo* assembly to increase continuity, accuracy, and completeness. The assembly includes 2.7 Gb, and is >250x more continuous than the original assembly, with contig N50 >25 Mb and L50 of 32. We also greatly expanded supporting RNA-based data for annotation that identifies 30,396 total genes (21,039 protein coding). The new reference assembly is accessible in annotated form for public use.

Keywords

Bovine genome, reference assembly, cattle, Hereford

Data Description

Context

There are an estimated 1.4 billion domesticated cattle (*Bos taurus*) in the world, being raised primarily for meat and dairy in a diversity of climates and production schemes[1]. This wide diversity of environments has led to the selection of individual breeds of cattle, as adaptation for specific needs is required to enhance efficiency and sustainability of production. Despite bottlenecks imposed by breed formation in the relatively recent past, there remains substantial

genetic variation within cattle populations that responds to selection for specific traits[2]. Selection progress has been enhanced by the use of genomic tools based on a cattle reference genome[3,4], especially in dairy cattle in the U.S. and Europe. The first bovine reference genome was created by a large consortium of researchers and funding institutions, led by the Human Genome Sequencing Center at Baylor College of Medicine. The prevailing methods of the time were improved by the use of inbreeding to decrease the contrast between parental alleles and consequent assembly problems, and by the use of a female to improve coverage of the X chromosome. A Hereford cow, L1 Dominette 01449 (Figure 1), whose sire was also her grandsire and who had an inbreeding coefficient of 0.30, was selected from the USDA Agriculture Research Service’s Livestock and Range Research Laboratory herd in Miles City, Montana, USA for creation of the reference assembly[5]. We report a new assembly for the same animal, to provide context for existing data created with the previous reference, but improved by over 200-fold in continuity and 10-fold in accuracy. We have also added extensive data to improve the annotation of genes and other genomic features. The new genome and annotation facilitate studies on improving cattle, which is a species of global economic relevance.

Methods

a) Genome sequencing

The original Hereford assembly used blood as the source of DNA, leading to difficulties in assembling specific genomic regions that undergo rearrangement in nucleated blood cells. Therefore, we used high molecular weight (HMW) genomic DNA extracted from frozen lung tissue as the source for the improved reference, supporting accurate assembly of regions that include important immune function loci. The HMW DNA was extracted and used to construct

libraries for SMRT sequencing as previously described[6]. Libraries were sequenced on a PacBio RS II with 318 cells of chemistry P6-C4 yielding 244 Gb (~80x coverage) of sequence (Table S1) with an average read length of 20 kb. Additional genomic DNA, also from frozen lung tissue, was used to construct two Illumina TruSeq PCR-free 2x150 bp paired-end libraries, LIB24773 with an average insert size of 450 bp and LIB18483 with an average insert size of 600 bp. The libraries were sequenced on an Illumina NextSeq500 with LIB24773 sequenced on one flow cell yielding 111 Gb and LIB18483 sequenced on two flow cells yielding 97.6 Gb and 131.3 Gb, respectively (Table S1).

b) Assembly, scaffolding and gap filling

PacBio long reads were assembled using the Falcon *de novo* genome assembler (version 0.4.0)[7]. A length cutoff of 10 kb was used for the initial seed read alignment, and a secondary cut of 8 kb for the pre-assembled reads before layout of the assembly. The assembly resulted in 3077 primary contigs covering 2.7 Gb with a contig N50 of 12 Mb (Figure 2). A single round of polishing the assembly was carried out to improve base accuracy[8]. Raw data was mapped back to the assembly using blasr[9], and a new consensus called with the Quiver algorithm, both carried out using the resequencing pipeline from the SMRT Analysis 3.1.1 software package (Pacific Biosciences, Menlo Park, CA).

Three data sets were used to scaffold contigs: Dovetail Chicago[10], BtOM1.0 optical map[11], and a recombination map developed by Ma *et al*.[12] (Figure 2). First, a Chicago library was prepared as described previously[10] from Dominette lung tissue and sequenced on an Illumina

HiSeq 2500 to approximately 84x coverage (LIB14630, Table S1). The Falcon assembly and Chicago library read pairs were used as input data for HiRise[10], a software pipeline for using Chicago data to scaffold genomes. The separations of Chicago read pairs mapped within contigs were analyzed by HiRise to produce a likelihood model for genomic distance between read pairs, and the model was used to identify putative misjoins and score prospective joins. After scaffolding, long reads were used to close gaps between contigs resulting in 2,511 scaffolds with an N50 of 63 Mb and L50 of 16. Next we used the Dominette derived *Bos taurus* optical map BtOM1.0[11] that spans 2,575.30 Mb and comprises 78 optical contigs to further scaffold the Dovetail assembly. The IrysView v2.5.1 software package (BioNano Genomics, San Diego, CA) was used to map the assembly scaffolds to the optical map contigs. After a manual curation step where false joins and misassembled contigs were detected by inspection of the alignment, IrysView scaffolding reduced the number of scaffolds to 50 while the scaffold L50 decreased to 12 and the scaffold N50 increased to 108 Mb. Finally, approximately 54k SNP markers from the bovine recombination map[12] were used to detect mis-assemblies and scaffold the 29 acrocentric autosomes[13,14]. Markers were aligned to the optical map scaffolds with BLAST[15] requiring 98% mapping identity over the full marker sequence length. Only unique mapping SNPs were considered. Scaffolds were broken when two or more markers from different linkage groups aligned to them. Pearson correlation coefficients between scaffold marker alignment order and genetic map marker order were used to calculate the most probable scaffold order and orientation. Another round of polishing was undertaken with Arrow with the SMRT Analysis 3.1.1 software package.

Gap filling was first done by aligning two Canu (Canu, RRID:SCR_015880) v1.4 [16] assemblies (run with different overlap algorithms implemented within Canu for error correction, MHAP[17] and minimap[18]) to the scaffolded assembly and identifying alignments crossing gaps. A gap was filled if either assembly spanned a gap with >5,000 bp aligning on either side of the gap up to at most 10 bp away from the gap. In the case of a negative gap (i.e. the assemblies had a collapse), both assemblies had to agree on the position and size of the collapse. In total, 171 gaps were closed with this approach. Finally, PBJelly (PBJelly, RRID:SCR_012091) pbsuite v.15.8.24 [19] was used to fill an additional 91 gaps. The closing of gaps between contigs increased the contig N50 from 12 Mb to 21 Mb and reduced the number of gaps in the genome to 459.

c) Manual curation

Following gap filling, the assembly was manually curated. To start, we assessed the X chromosome using two assemblies produced from MaSuRCA (MaSuRCA, RRID:SCR_010691) [20] error-corrected reads (PacBio corrected with Illumina). The first used Canu v1.4 to assemble the MaSuRCA corrected reads and the other used Celera Assembler[17] version 8.3. MUMmer 3.0[21] alignments between these two assemblies and the gap-filled assembly were used to confirm or revise the order and orientation of X-chromosome contigs as well as place additional unplaced contigs and scaffolds. Next, the autosomal assembly structure was manually curated and oriented with an independent genetic map UMCLK (Table S2, Supplementary Note). The BLAT (BLAT, RRID:SCR_011919) alignment tool[22] and BWA MEM (BWA, RRID:SCR_010910) [23] were used to map the probe and flanking sequences present on

commercially available genotyping assays to identify misassemblies. Assembly gaps, Illumina read-depth coverage and alignments with dbSNP sequences and flanking sequences were used to refine breakpoints for sequence rearrangements using a combination of custom scripts in iterative fashion[24]. In all, corrections were made to chromosomes 1, 2, 5-12, 16, 18-21, 23, 26, 27, and X. PBJelly was run on the curated assembly to close remaining gaps. The number of gaps decreased from 459 to 386, indicating that our manual curation correctly oriented contigs such that PBJelly could now fill an additional 73 gaps that could not previously be filled. The remaining gaps represent regions where either the gap is too large for our PacBio reads to span, read coverage is low or missing, or there is a remaining misassembly. The contig N50 also increased again from 21 Mb to 26 Mb. Polishing of the assembly proceeded through one iteration of Arrow with all the raw PacBio reads followed by polishing with short Illumina reads (SRR2226514 and SRR2226524 as well as LIB24773 and one run, 97.6 Gb, of LIB18483) using Pilon (Pilon, RRID:SCR_014731) v1.22 [25] with the parameters "--diploid --fix indels --nostrays". The final version of the genome (ARS-UCD1.2) contains 2,628,394,923 bp on the 30 chromosomes (Figure 2b) with an additional 87.5 Mb of unplaced sequence and is available from NCBI under the accession GCF_002263795.1.

d) RNA sequencing

The Iso-Seq method for sequencing full-length transcripts was developed by Pacific Biosciences during the same time period as the genome assembly. We, therefore, employed this technique to improve characterization of transcript isoforms expressed in cattle tissues using a diverse set of tissues collected from L1 Dominette 01449 upon euthanasia. The data were collected using an

early version of the Iso-Seq library protocol[26] as suggested by Pacific Biosciences. Briefly, RNA was extracted from each tissue using Trizol reagent as directed (Thermofisher). Two micrograms of RNA were then selected for PolyA tails, and converted into cDNA using the SMARTer PCR cDNA Synthesis Kit (Clontech). The cDNA was amplified in bulk with 12-14 rounds of PCR in eight separate reactions, then pooled and size-selected into 1-2 kb, 2-3 kb, and 3-6 kb fractions using the BluePippin instrument (Sage Science). Each size fraction was separately re-amplified in eight additional reactions of 11 PCR cycles. The products for each size fraction amplification were pooled and purified using AMPure PB beads (Pacific Biosciences) as directed, and converted to SMRTbell libraries using the Template Prep Kit v1.0 (Pacific Biosciences) as directed. Iso-Seq was conducted for 22 tissues including abomasum, aorta, atrium, cerebral cortex, duodenum, hypothalamus, jejunum, liver, longissimus dorsi muscle, lung, lymph node, mammary gland, medulla oblongata, omasum, reticulum, rumen, subcutaneous fat, temporal cortex, thalamus, uterine myometrium, and ventricle from the reference cow as well as the testis of her sire. The size fractions were sequenced in either four (for the smaller two fractions) or five (for the largest fraction) SMRTcells on the RSII instrument. Isoforms were identified using the Cupcake ToFU pipeline[27] without using a reference genome.

Short-read based RNA-seq data derived from tissues of Dominette were available in the GenBank database, as her tissues have been a freely-distributed resource for the research community. To complement and extend these data, and to ensure that the tissues used for Iso-Seq were also represented by RNA-seq data for quantitative analysis and confirmation of isoforms observed in Iso-Seq, we generated additional data avoiding overlap with existing public data.

Specifically, the TruSeq stranded mRNA LT kit (Illumina, Inc) was used as directed to create RNA-seq libraries, which were sequenced to a minimum of 30 million reads for each tissue sample. The Dominette tissues that were sequenced in this study include abomasum, anterior pituitary, aorta, atrium, bone marrow, cerebellum, duodenum, frontal cortex, hypothalamus, KPH fat (internal organ fat taken from the covering on the kidney capsule), lung, lymph node, mammary gland (lactating), medulla oblongata, nasal mucosa, omasum, reticulum, rumen, subcutaneous fat, temporal cortex, thalamus, uterine myometrium, and ventricle. RNA-seq libraries were also sequenced from the testis of her sire. All public datasets, and the newly sequenced RNA-seq and Iso-Seq datasets, were used to annotate the assembly, to improve the representation of low-abundance and tissue-specific transcripts and to properly annotate potential tissue-specific isoforms of each gene.

e**) Annotation**

The NCBI Eukaryotic Genome Annotation Pipeline was used to annotate genes, transcripts, proteins and other genomic features on ARS-UCD1.2. Nearly 13 billion RNA-seq reads from over 50 tissues and 553,798 consensus Iso-Seq reads from 23 tissues were retrieved from SRA (Table S1) and aligned to the masked genome, along with 12,472 known RefSeq transcripts, 19,820 GenBank transcripts, and 1,583,270 ESTs, using BLAST[15] followed by Splign[28]. The set of proteins aligned to the masked genome consisted of 13,381 RefSeq proteins and 16,371 GenBank proteins from cattle, and 50,089 RefSeq proteins from human. The gene models' structures and boundaries were primarily derived from these alignments. Where alignments did not define a complete model but the coding propensity of the region was sufficiently high, *ab initio* extension or joining/filling of partial ORFs in compatible frame was performed by Gnomon[29], using a hidden Markov model trained on cattle. tRNAs were

predicted with tRNAscan-SE (tRNAscan-SE, RRID:SCR_010835) 1.23[30] and small non-coding RNAs were predicted by searching the RFAM 12.0 HMMs for eukaryotes using cmsearch from the Infernal (Infernal, RRID:SCR_011809) package[31]. The annotation of the ARS-UCD1.2 assembly, Annotation Release 106 (AR 106[32]) resulted in 21,039 protein-coding genes, 9,357 non-coding genes and 4,569 pseudogenes.

The respective quality of the UMD3.1.1 annotation (Annotation Release 105 AR 105[33]) and AR 106 was evaluated by aligning the annotated proteins of each release to the UniProtKB/SwissProt proteins available in Entrez Protein (returned by the Entrez query srcdb_swiss_prot[properties] AND eukaryotes[orgn] on 7/29/2019) using BlastP. For each protein coding gene, the protein isoform with the best alignment based on score (or in case of a tie, based on alignment length, percent coverage or subject protein length) was chosen as the isoform representative of the gene. The counts of protein coding genes in AR 105 and AR 106 with representative isoforms covering at least 95% of the length of the UniProtKB/SwissProt proteins were then compared.

Data Validation and quality control

Quality assessment

To assess the error profile of our assembly and compare it to the previous reference, UMD3.1.1[34], (NCBI accession GCF_000003055.5) long- and short-read sequences from Dominette were aligned to both assemblies. Short-read BWA alignments of LIB18483 sequences not used for polishing were evaluated from feature response curves computed with FRCbam[35]

(Figure 3a). The total number of erroneous features in ARS-UCD1.2 decreased by over 20% compared to UMD3.1.1 (Table 1). Errors on the chromosome scaffolds exhibited a > 40% reduction in error features compared to UMD3.1.1, suggesting that ARS-UCD1.2 chromosomes were better representative of the individual sequenced. The error classes most prevalent on the ARS-UCD1.2 unplaced sequences compared to the chromosomes were HIGH COV PE, HIGH NORM COV PE, and HIGH SPAN PE with unplaced sequences accounting for 73%, 80%, and 65% of the errors in each class respectively. The increased percentage of HIGH COV PE and HIGH NORM COV PE errors indicates that many of the unplaced sequences are over-assembled or collapsed while HIGH SPAN PE errors would be expected as the majority of the 2,181 unplaced sequences are fragmented. The same short-read alignments were also used to estimate the quality value (QV) of the assembly with ARS-UCD1.2 scoring 48.67 and UMD3.1.1 37.98, which correspond to a per-base error rate of 1.58×10^{-5} and 1.59×10^{-4}, respectively, or an order-of-magnitude improvement in accuracy. This was calculated from the number of non-matching base calls from FreeBayes (FreeBayes, RRID:SCR_010761)[36] as previously described[6]. UMD3.1.1's lower per-base accuracy resulted from the large number of gaps in the assembly, the larger proportion of unplaced contigs and the incomplete resolution of larger repetitive regions.

As a measure of the completeness of the assemblies and to define the chromosome ends, we identified centromeric[24] and telomeric[37] repeats (Table S3). For the 29 acrocentric autosomes, we identified the expected centromeric and telomeric repeats on 9 ARS-UCD1.2 chromosomes (5,6,8,10,13,14,16,17, and 18) whereas no UMD3.1.1 chromosomes contained both, mainly due to a relative lack of telomeric repeats in the assembly. ARS-UCD1.2 chromosomes 3,20, and 22 are missing both chromosome ends, while chromosomes 1,9,10, and

15 erroneously contain centromeric repeats at both ends. Finally, the metacentric X chromosome only has telomeric repeats at one end and no centromeric repeats. Telomeric repeats were only identified on UMD3.1.1 chromosome 20, centromeric repeats are found on the proper end of 22 autosomes (missing on 6,7,20,21,22,27, and 28) and the X chromosome contains centromeric repeats. All chromosomes also contain centromeric repeats dispersed throughout so it is difficult to determine if the X centromere is properly placed. Centromeric repeat regions at the start of ARS-UCD1.2 chromosome scaffolds were over 2-fold larger than their counterparts in the UMD3.1 reference (Figure S1). In order to further assess the structural integrity of both assemblies, we used Sniffles[38] to evaluate the concordance of long reads from Dominette on both assemblies. All SV classes showed sharp declines in prevalence in ARS-UCD1.2 vs UMD3.1.1 (Table 1). Deletions, duplications, insertions, and inversions all declined by at least 98%.

Table 1. Assembly quality score value statistics and structural inconsistencies measured between ARS-UCD1.2 and UMD3.1.1 using Dominette WGS reads.

Major Category	Sub Category	ARS-UCD1.2	UMD3.1.1	Description
QV		48.67	37.98	Quality value estimate (Phred-scale)
FRCbam				
	COMPR PE	37,309 (30,643)[1]	54,602 (52,606)	Areas with low Compression/Expansion statistics
	STRECH PE	37,255 (22,741)	35,766 (35,299)	Areas with high CE statistics
	HIGH COV PE	7166 (1970)	7711 (6331)	High read coverage areas (all aligned reads)
	HIGH NORM COV PE	5641 (1125)	7109 (5778)	High paired-read coverage areas (only properly aligned pairs)

	HIGH OUTIE PE	139 (102)	2108 (2108)	Regions with high numbers of misoriented or distant pairs
	HIGH SINGLE PE	60 (53)	1258 (1256)	Regions with high numbers of unmapped pairs
	HIGH SPAN PE	4882 (1687)	4172 (3582)	Regions with high numbers of pairs that map to different scaffolds
	LOW COV PE	43,370 (36,062)	57,176 (56,648)	Low read coverage areas (all aligned reads)
	LOW NORM COV PE	42,067 (34,592)	60,560 (59,926)	Low paired-end coverage areas (only properly aligned pairs)
	Total Features	177,889 (128,975)	230,462 (223,534)	All erroneous features
Sniffles[2]				
	DEL	188	10504	Deletions
	DUP	16	728	Duplications
	INS	106	4911	Insertions
	INV	34	2675	Inversions
	Total SVs	344	18,818	All structural variants

[1] Numbers in parentheses indicate the errors in placed chromosomes scaffolds only.

[2] Sniffles structural variant (SV) calls were generated using long-reads aligned to the whole assembly.

Improved contiguity

A key measure of improvement over the previous reference is the increase in the contiguity of the genome (Figure 2). The 30 cattle chromosomes are now composed of 345 contigs compared to 72,264 contigs in the UMD3.1.1 assembly. This represents a 280-fold increase in the contig NG50 (N50 calculated from a fixed 2.8Gb genome size), from 0.092 Mb to 25.8 Mb (Figure 3b) and a 209-fold increase in sequence continuity. The 345 contigs in ARS-UCD1.2 equate to 315 gaps in the chromosomes vs. 72,234 on UMD3.1.1. We demonstrated the impact of higher contiguity on the mapping of existing datasets by aligning the currently-available 14,473 known cattle RefSeq transcripts (a manually curated set of transcript accessions prefixed with NM_ and

NR_) to both ARS-UCD1.2 and UMD3.1.1. We found that the transcripts aligned more cleanly to ARS-UCD1.2 than to UMD3.1.1 (Table 2). The number of transcripts for which the best alignment covered less than 95% of the CDS went down from 734 on UMD3.1.1 to only 37 for ARS-UCD1.2. Moreover, the alignment of 219 transcripts were split across two or more genomic sequences of UMD3.1.1 compared to only 9 for ARS-UCD1.2. Although a greater number of transcripts failed to align to ARS-UCD1.2, this difference is made up of transcripts from Y-linked genes (Table S4). The presence of Y-linked genes in the UMD3.1.1 assembly is likely due to Y chromosome contamination from the inclusion of sequence from a bacterial artificial chromosome library prepared from Dominette's sire [34,39]. Since ARS-UCD1.2 is derived from an XX female and does not contain the Y chromosome, we recommend the inclusion of an independently assembled Y chromosome prior to analysis as is being done by the 1000 Bull Genomes Project [40].

Table 2: Splign alignment of RefSeq transcripts to ARS-UCD1.2 and UMD3.1.1

Name	ARS-UCD1.2	UMD3.1.1
Accession	GCF_002263795.1	GCF_000003055.5
Number of sequences retrieved from Entrez	14,473	14,473
Number of sequences not aligning[1]	19 (12)	13 (12)
Number of sequences whose best alignments span multiple loci (split genes)	9	219
Number of sequences with CDS coverage < 95%	37	734

[1]Neither assembly includes a Y chromosome yet 7 transcripts (6 not aligning to only ARS-UCD1.2 and 1 not aligning to both) are from Y-linked genes. Totals excluding Y-linked genes in parenthesis.

Annotation comparison

The ARS-UCD1.2 assembly annotation (AR 106) generated by NCBI was compared to the UMD3.1.1 annotation (AR 105). About 2/3 of the genes (85% of protein-coding genes) are identical or nearly identical (with a support score, derived from a combination of matching exon boundaries and sequence overlap, of 0.66 or more, on a scale of 0 to 1, on both sides of the

comparison) between the two datasets (Table S5). Over 90% of the novel genes (19% of total genes) in AR 106 were non-coding genes, due in part to the addition of a module for the prediction of short non-coding genes based on RFAM models to the annotation pipeline after AR 105 was produced. The number of protein-coding genes with at least one isoform covering 95% of the length of a UniProt/SwissProtKB protein is 17,810 (85% of protein-coding genes) for AR 106 versus 16,956 (80%) for AR 105, suggesting that the protein models predicted in AR 106 are generally more complete than in AR 105.

These improvements in the annotation are partly due to the availability of more and longer transcript evidence for gene prediction (Iso-Seq in particular), but it is clear that uncertainty of placement and orientation of sequence across gaps has a large impact on gene annotation. Of the 21,039 genes annotated in ARS-UCD1.2, 69 (0.3%) have gaps within introns compared to 6,949 (33%) of annotated UMD3.1.1 genes (Figure 3c). Considering the potential impact of regulatory elements flanking genes, it is also important to note that almost 60% of UMD3.1.1 genes have gaps within 10 kb while that percentage drops below 1% in ARS-UCD1.2.

ARS-UCD1.2 also represents an improvement in base accuracy over UMD3.1.1 that is measurable in the annotation. High rates of sequencing error can disrupt the prediction of open reading frames and lead to truncated gene models or the erroneous calling of non-coding genes or pseudogenes instead of protein-coding genes. The NCBI annotation process attempts to compensate for this problem by producing a ‘corrected’ model (with name prefixed with LOW QUALITY) containing a difference with the genome sequence, when protein alignments suggest there is an erroneous indel in the genome. The number of such ‘corrected’ models decreased by 44% from 1,828 in UMD3.1.1/AR 105 to 1,027 in ARS-UCD1.2/AR 106.

Conclusions

This assembly represents a 200-fold improvement in sequence continuity and a 10-fold improvement in per-base accuracy over previous cattle assemblies. The assignment of megabase-length contigs to full chromosome scaffolds provides additional certainty in gene and genetic marker positions which will influence marker-assisted selection and basic research. The assembly was selected as the reference genome for taurine cattle by the US genomic evaluation system in December 2018[41] and the 37 partner institutions of the 1000 Bull Genomes Project for the run7 variant calls distributed globally in June 2019[40]. We demonstrate that assembly improvements warranted adoption by these projects and that increased assembly accuracy will benefit future genetics research on this species.

Availability of supporting data and materials

Accession numbers for raw sequencing reads and assemblies can be found in Table S1. Supporting data is also available through a GigaDB dataset[42].

Additional files

Table S1. Sequencing resources

Table S2. UMCLK genetic map

Table S3. Centromeric and telomeric repeats

Table S4. RefSeq transcripts not aligning to assemblies

Table S5. ARS-UCD1.2 UMD3.1.1 annotation comparison

Figure S1. Average Centromeric Repeat regions. Centromeric satellite regions identified by RepeatMasker in the ARS-UCD1.2 (ARSUCD) and UMD3.1.1 (UMD3) assemblies were merged if they overlapped by one bp. Histogram bars show the average length of these regions that are within the first 500 kb of a chromosome scaffold’s starting base (CHRSTART), within

unplaced scaffolds (UNPLACED) or in the middle of the chromosome scaffolds (CHRSCAFF). Error bars represent the 95% confidence interval (two standard errors from the mean) of centromere lengths in each category.

Supplemental Note. UMCLK genetic map

Abbreviations

AR: annotation release; bp: base pairs; BWA: Burrows-Wheeler Aligner; Gb: gigabase pairs; HMW: high molecular weight; kb: kilobase pairs; Mb: megabase pairs; NCBI: National Center for Biotechnology Information; RefSeq: NCBI Reference Sequence Database; RNA-seq: high-throughput short-read messenger RNA sequencing; SMRT: single molecule real-time; SNP: single-nucleotide polymorphism; SRA: Sequence Read Archive; SV: structural variant; tRNA: transfer RNA

Competing interests

RH and ET are employed by Pacific Biosciences, all other authors declare that they have no competing interests.

Author contributions

TPLS, JFM, CPVT, RDS, DMB, and BDR conceived, initiated, and managed the project. TPLS and JFM were responsible for DNA and RNA sequence data production. BDR, DMB, RDS, SJS, CD, AZ, RH, JG, AR, SK, and AMP performed assembly and associated tasks. TNR, WYL, CC, WL, SDM, BMM, WMS, JAH, JCS, WN, SGS, JBC, GL, and CPVT performed quality control and/or contributed additional analyses. CGE, TGM, and ET performed RNA analyses. FTN and JH performed annotation and managed public presentation of the assembly files. All authors read and edited the manuscript.

Acknowledgements

Sequence generation was supported by USDA/NRSP-8 Animal Genome, USDA-ARS Meat Animal Research Center, Neogen, and Zoetis. BDR, SGS, JBC, CPVT, and GL were supported by USDA CRIS 8042-31000-001-00-D. JBC was supported by USDA CRIS 8042-31000-002-00-D. DMB and WL were supported in part by USDA CRIS 5090-31000-026-00-D. DMB was also supported in part by USDA NIFA grant 5090-31000-026-06-I. WMS, TGM, TPLS were supported by USDA CRIS 3040-31000-100-00-D. RDS and TNR were supported in part by USDA NIFA 2016-68004-24827. RDS was also supported in part by USDA NIFA 2013-67015-21202, 2015-67015-23183, NIH 1R01HD084353-01A1 and USDA Hatch MO-HAAS0001. JH and FTN were supported by the Intramural Research Program of the National Library of Medicine, National Institutes of Health. JAH and JCS were supported by funding from the UKRI-BBSRC awards BB/M027155/1, BBS/E/I/00007035, BBS/E/I/00007038 and BBS/E/I/00007039. SK, AR, and AMP were supported by the Intramural Research Program of the National Human Genome Research Institute, National Institutes of Health, AR was also supported by the Korean Visiting Scientist Training Award (KVSTA) through the Korea Health Industry Development Institute (KHIDI), funded by the Ministry of Health & Welfare (HI17C2098). This work utilized the computational resources of the NIH HPC Biowulf cluster (https://hpc.nih.gov). Mention of trade names or commercial products in this article is solely for the purpose of providing specific information and does not imply recommendation or endorsement by the US Department of Agriculture.

References

1. Robinson, T. P. *et al.* Mapping the Global Distribution of Livestock. *PLOS ONE* **9**, e96084 (2014).

2. Weigel, K. A., VanRaden, P. M., Norman, H. D. & Grosu, H. A 100-Year Review: Methods and impact of genetic selection in dairy cattle—From daughter–dam comparisons to deep learning algorithms. *J. Dairy Sci.* **100**, 10234–10250 (2017).
3. Saatchi, M., Schnabel, R. D., Rolf, M. M., Taylor, J. F. & Garrick, D. J. Accuracy of direct genomic breeding values for nationally evaluated traits in US Limousin and Simmental beef cattle. *Genet. Sel. Evol.* **44**, 38 (2012).
4. García-Ruiz, A. *et al.* Changes in genetic selection differentials and generation intervals in US Holstein dairy cattle as a result of genomic selection. *Proc. Natl. Acad. Sci. U. S. A.* **113**, E3995–E4004 (2016).
5. Bovine Genome Sequencing and Analysis Consortium, Elsik, C. G., Tellam, R. L. & Worley, K. C. The Genome Sequence of Taurine Cattle: A Window to Ruminant Biology and Evolution. *Science* **324**, 522–528 (2009).
6. Bickhart, D. M. *et al.* Single-molecule sequencing and chromatin conformation capture enable de novo reference assembly of the domestic goat genome. *Nat. Genet.* **49**, 643–650 (2017).
7. Chin, C.-S. *et al.* Phased diploid genome assembly with single-molecule real-time sequencing. *Nat. Methods* **13**, 1050–1054 (2016).
8. Chin, C.-S. *et al.* Nonhybrid, finished microbial genome assemblies from long-read SMRT sequencing data. *Nat. Methods* **10**, 563–569 (2013).
9. Chaisson, M. J. & Tesler, G. Mapping single molecule sequencing reads using basic local alignment with successive refinement (BLASR): application and theory. *BMC Bioinformatics* **13**, 238 (2012).

10. Putnam, N. H. *et al.* Chromosome-scale shotgun assembly using an in vitro method for long-range linkage. *Genome Res.* **26**, 342–350 (2016).

11. Zhou, S. *et al.* A clone-free, single molecule map of the domestic cow (Bos taurus) genome. *BMC Genomics* **16**, 644 (2015).

12. Ma, L. *et al.* Cattle Sex-Specific Recombination and Genetic Control from a Large Pedigree Analysis. *PLOS Genet.* **11**, e1005387 (2015).

13. KHP-Informatics/illumina-array-protocols. *GitHub* Available at: https://github.com/KHP-Informatics/illumina-array-protocols. (Accessed: 6th September 2019)

14. Iannuzzi, L. & Di Meo, G.P. Chromosomal evolution in bovids: a comparison of cattle, sheep and goat G- and R-banded chromosomes and cytogenetic divergences among cattle, goat and river buffalo sex chromosomes. Chromosome Res. **3**, 291–299 (1995).

15. Altschul, S. F., Gish, W., Miller, W., Myers, E. W. & Lipman, D. J. Basic local alignment search tool. *J. Mol. Biol.* **215**, 403–410 (1990).

16. Koren, S. *et al.* Canu: scalable and accurate long-read assembly via adaptive k-mer weighting and repeat separation. *Genome Res.* gr.215087.116 (2017). doi:10.1101/gr.215087.116

17. Berlin, K. *et al.* Assembling large genomes with single-molecule sequencing and locality-sensitive hashing. *Nat. Biotechnol.* **33**, 623–630 (2015).

18. Li, H. Minimap and miniasm: fast mapping and de novo assembly for noisy long sequences. *Bioinformatics* **32**, 2103–2110 (2016).

19. English, A. C. *et al.* Mind the Gap: Upgrading Genomes with Pacific Biosciences RS Long-Read Sequencing Technology. *PLOS ONE* **7**, e47768 (2012).

20. Zimin, A. V. *et al.* Hybrid assembly of the large and highly repetitive genome of Aegilops tauschii, a progenitor of bread wheat, with the MaSuRCA mega-reads algorithm. *Genome Res.* **27**, 787–792 (2017).

21. Kurtz, S. *et al.* Versatile and open software for comparing large genomes. *Genome Biol.* **5**, R12 (2004).

22. Kent, W. J. BLAT—The BLAST-Like Alignment Tool. *Genome Res.* **12**, 656–664 (2002).

23. Li, H. Aligning sequence reads, clone sequences and assembly contigs with BWA-MEM. *ArXiv13033997 Q-Bio* (2013).

24. Bickhart, D. *Scripts and documentation related to the assembly of ARS-UCD1.2: https://github.com/njdbickhart/CattleAssemblyScripts*. (2019).

25. Walker, B. J. *et al.* Pilon: An Integrated Tool for Comprehensive Microbial Variant Detection and Genome Assembly Improvement. *PLOS ONE* **9**, e112963 (2014).

26. Procedure & Checklist - Isoform Sequencing (Iso-Seq™) using the Clontech SMARTer PCR cDNA Synthesis Kit and Manual Agarose-gel. 16

27. Tseng, E. *Miscellaneous collection of Python and R scripts for processing Iso-Seq data: https://github.com/Magdoll/cDNA_Cupcake*. (2019).

28. Kapustin, Y., Souvorov, A., Tatusova, T. & Lipman, D. Splign: algorithms for computing spliced alignments with identification of paralogs. *Biol. Direct* **3**, 20 (2008).

29. Gnomon - the NCBI eukaryotic gene prediction tool. Available at: https://www.ncbi.nlm.nih.gov/genome/annotation_euk/gnomon/. (Accessed: 8th August 2019)

30. Lowe, T. M. & Eddy, S. R. tRNAscan-SE: A Program for Improved Detection of Transfer RNA Genes in Genomic Sequence. *Nucleic Acids Res.* **25**, 955–964 (1997).

31. Nawrocki, E. P. *et al.* Rfam 12.0: updates to the RNA families database. *Nucleic Acids Res.* **43**, D130–D137 (2015).
32. Bos taurus Annotation Report 106. Available at: https://www.ncbi.nlm.nih.gov/genome/annotation_euk/Bos_taurus/106/. (Accessed: 15th August 2019)
33. Bos taurus Annotation Report 105. Available at: https://www.ncbi.nlm.nih.gov/genome/annotation_euk/Bos_taurus/105/. (Accessed: 15th August 2019)
34. Zimin, A.V. *et al.* A whole-genome assembly of the domestic cow, Bos taurus. Genome Biology **10**, R42 (2009).
35. Vezzi, F., Narzisi, G. & Mishra, B. Reevaluating Assembly Evaluations with Feature Response Curves: GAGE and Assemblathons. *PLOS ONE* **7**, e52210 (2012).
36. Garrison, E. & Marth, G. Haplotype-based variant detection from short-read sequencing. *ArXiv12073907 Q-Bio* (2012).
37. Koren, S. & Rhie, A. Vertebrate Genomes Project repository for the genome assembly working group find_telomere. https://github.com/VGP/vgp-assembly/tree/master/pipeline/telomere. (Accessed: 24th January 2020)
38. Sedlazeck, F. J. *et al.* Accurate detection of complex structural variations using single-molecule sequencing. *Nat. Methods* **15**, 461 (2018).
39. Snelling, W.M. *et al*. A physical map of the bovine genome. Genome Biol **8**, R165 (2007).
40. 1000 Bull Genomes Project. Available at: http://www.1000bullgenomes.com/. (Accessed: 6th September 2019)

41. Null, D. J., VanRaden, P. M., Rosen, B. D., O'Connell, J. R. & Bickhart, D. M. Using the ARS-UCD1.2 reference genome in U.S. evaluations. *Interbull Bull* **55**:30–34 (2019).
42. Rosen B.D. *et al*. Supporting data for "De novo assembly of the cattle reference genome with single-molecule sequencing" GigaScience Database. 2019. http://dx.doi.org/10.5524/100669

Figures

Figure 1. L1 Dominette 01449. *The line bred Hereford cow was selected as the original cattle reference animal for her high level of inbreeding.*

Figure 2. Dominette* de novo *assembly. *A) Assembly pipeline. N50 is the minimum scaffold/contig length needed to cover 50% of the genome. L50 is the number of contigs required to reach N50. B) Cattle chromosomes painted with assembled contigs. A color shift indicates the switch from one contig to the next or the end of an alignment block. The left half of each chromosome shows UMD3.1.1 contigs while the right shows ARS-UCD1.2. To be conservative, contigs were ordered by UMD3.1.1 assembly positions, where there are conflicts in order between ARS-UCD1.2 and UMD3.1.1, the plot will display a color switch in ARS-UCD1.2. *Within scaffolds assigned to chromosomes*

Figure 3. Assembly assessments computed for ARS-UCD1.2 and UMD3.1.1. *A) Feature response curves computed for ARS-UCD1.2 and UMD3.1.1. B) Calculated NG showing a 280-fold increase of ARS-UCD1.2 in comparison to UMD3.1.1. C) The percentage of gaps in gene flaking regions are reduced from 33% to 0.3% in ARS-UCD1.2 in comparison to UMD3.1.1.*

Figure1

Click here to access/download;Figure;Figure1.tif

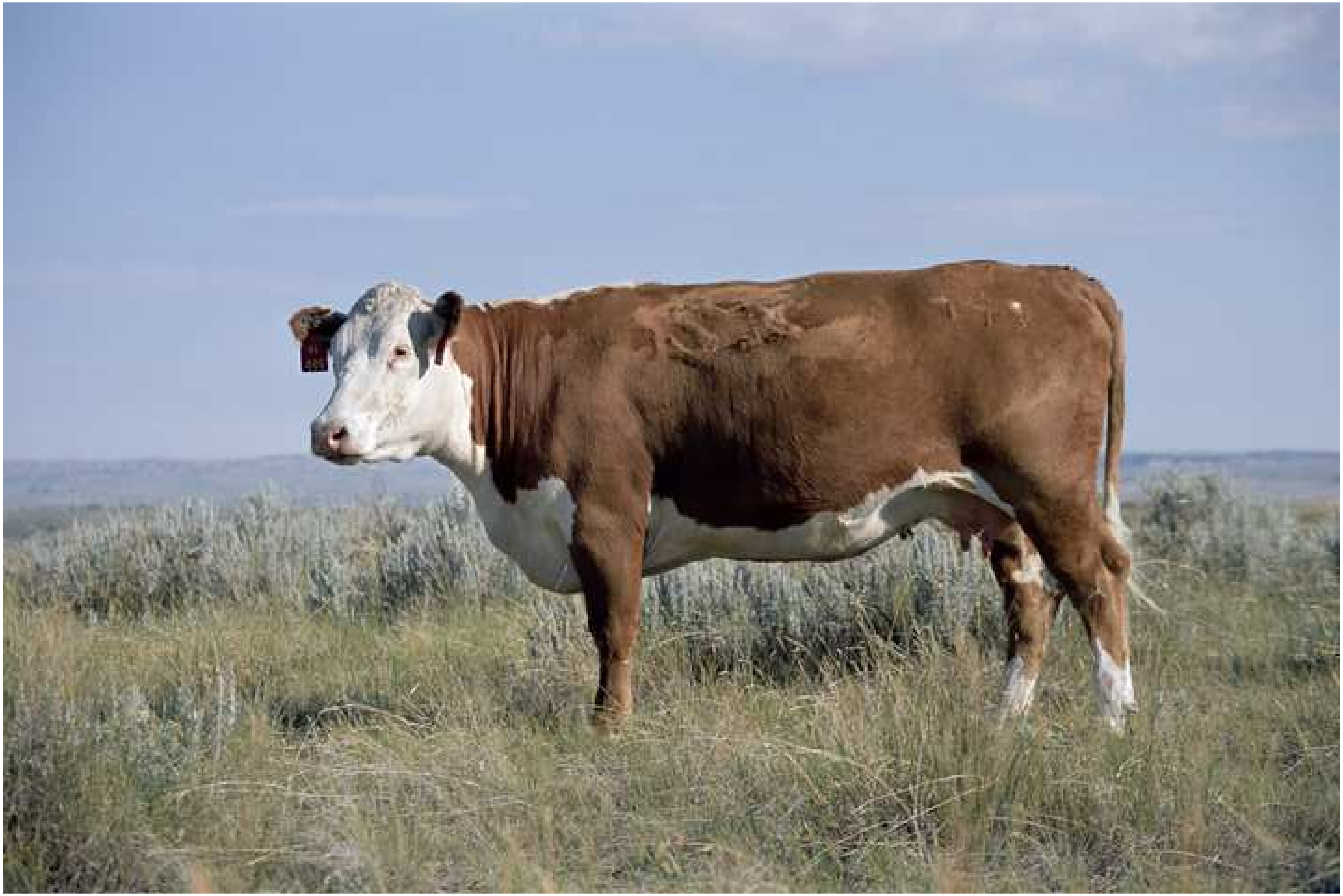

Figure2

Click here to access/download;Figure;Figure2.bmp

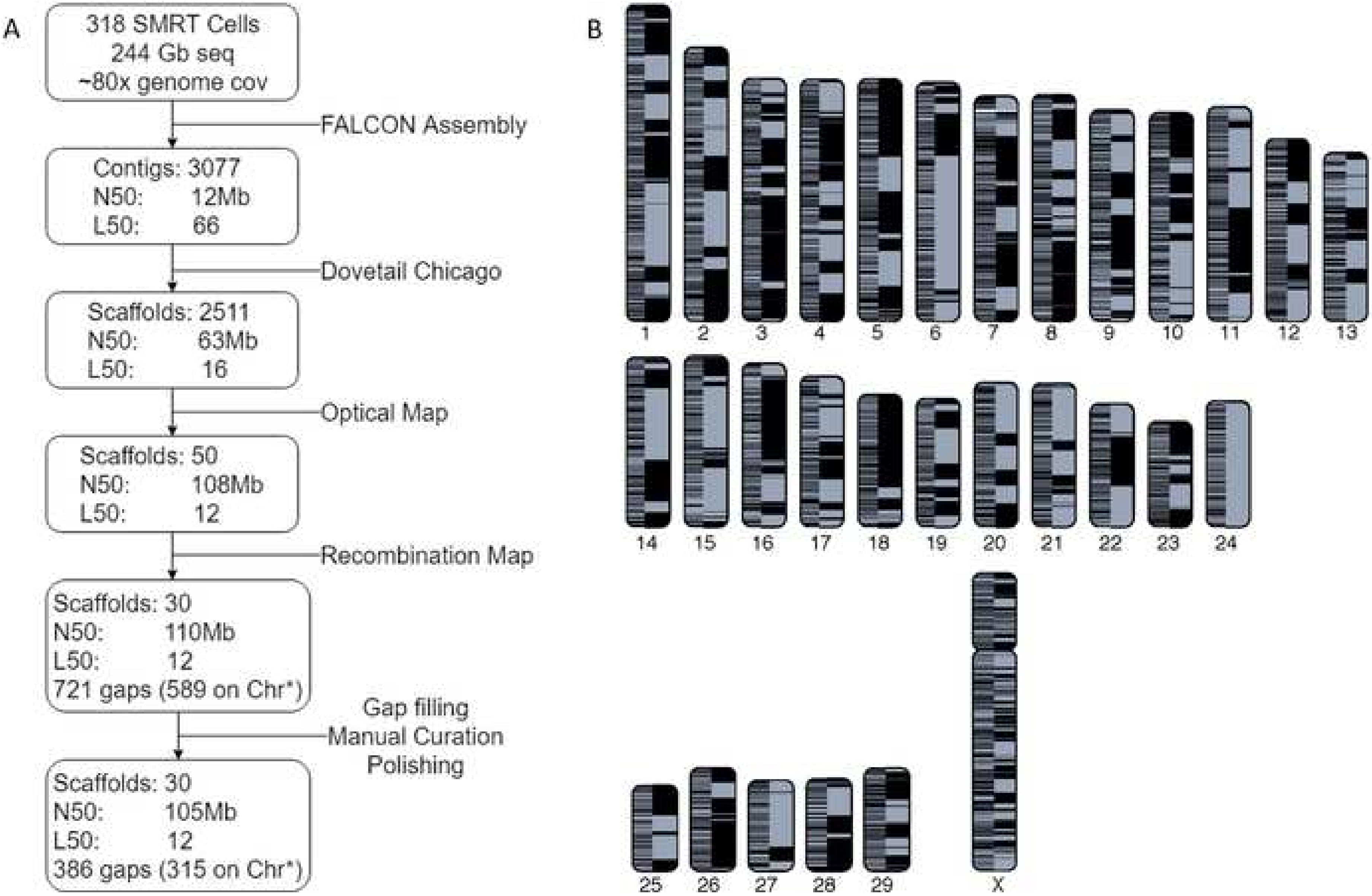

Figure3

Click here to access/download;Figure;Fig3.bmp

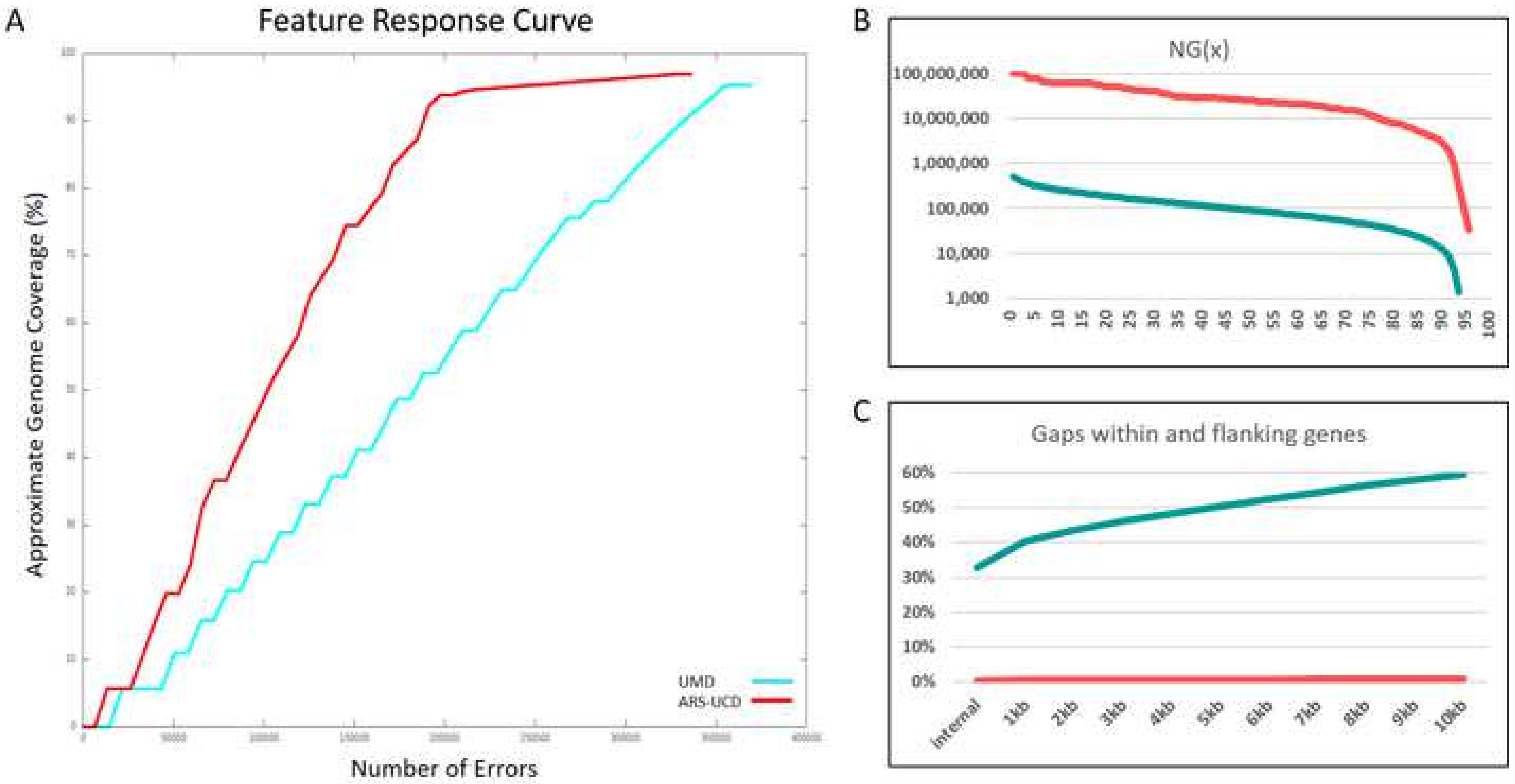

Click here to access/download
Supplementary Material
SupplementalNote_UMCLK_genetic_map.docx

Supplementary Table S1

Click here to access/download
Supplementary Material
TableS1_sequencing_resources.xlsx

Supplementary Table S3

Click here to access/download
Supplementary Material
TableS3_centromeric_and_telomeric_repeats.xlsx

Supplementary Table S4

Click here to access/download
Supplementary Material
TableS4_RefSeq_transcripts_not_aligning.xlsx

Supplementary Table S5

Click here to access/download
Supplementary Material
TableS5_annotation_comparison.xlsx

Personal Cover

Click here to access/download;Personal Cover;Response_to_reviewers.docx

Hans Zauner, PhD
Assistant Editor
GigaScience

RE: GIGA-D-19-00331

Dear Dr. Zauner,

We are grateful to you and the reviewers for evaluating our manuscript. We appreciate the reviewers for their comments and have implemented their suggestions. The revised manuscript is enhanced in meaningful ways. Our replies are in bold below and the text added to the manuscript is in italics. I have also included a photo of the sequenced individual as requested.

Sincerely,
Benjamin D. Rosen
Research Biologist (Computational), ARS, USDA
Animal Genomics and Improvement Laboratory
Building 306, Room 112, BARC-East
10300 Baltimore Ave
Beltsville, MD 20705-2350

Reviewer #1:

Specific comments for revision:

1. It is not clear from section (e) of the Methods how the alignments with UniProt/SwissProtKB were generated (i.e. through BLAST, Splign, or another tool).

REPLY: Text added to the Methods section (e).

The respective quality of the UMD3.1.1 annotation (Annotation Release 105 AR 105[33]) and AR 106 was evaluated by aligning the annotated proteins of each release to the UniProtKB/SwissProt proteins available in Entrez Protein (returned by the Entrez query srcdb_swiss_prot[properties] AND eukaryotes[orgn] on 7/29/2019) using BlastP. For each protein coding gene, the protein isoform with the best alignment based on score (or in case of a tie, based on alignment length, percent coverage or subject protein length) was chosen as the isoform representative of the gene. The counts of protein coding genes in AR 105 and AR 106 with representative isoforms covering at least 95% of the length of the UniProtKB/SwissProt proteins were then compared.

2. Related to this analysis, which release of UniProt/SwissProtKB was used?

REPLY: Date of access added to new text in Methods section (e).

(returned by the Entrez query srcdb_swiss_prot[properties] AND eukaryotes[orgn] on 7/29/2019)

3. Are protein sequences in the UniProt/SwissProtKB data set potentially derived in part from AR 105 or AR 106? Does this complicate interpretation of these results?

REPLY: There is a possibility that some of the proteins in the UniProt/SwissProtKB data set are from AR 105 or AR 106. However, Refseq sequences are not submitted to the International Nucleotide Sequence Database Collaboration and according to the UniProt documentation (https://www.uniprot.org/help/sequence_origin) "More than 95% of the protein sequences provided by UniProtKB come from the translations of coding sequences (CDS) submitted to the EMBL-

Bank/GenBank/DDBJ nucleotide sequence resources (International Nucleotide Sequence Database Collaboration", so the chances are small. The RefSeq proteins that would be incorporated into UniProt/SwissProtKB would have been manually curated and presumably have very strong support from experimental evidence. Whether or not RefSeq proteins are part of the UniProt/SwissProtKB, the strength of the analysis relies in the difference in the number of good hits between the two annotation releases rather than in the absolute numbers of hits for each release.

4. In the 'Annotation comparison' section, the authors state that "About 2/3 of the genes (85% of protein-coding genes) are identical or nearly identical between the two datasets." What qualifies as nearly identical?
REPLY: Nearly identical genes are highly similar genes, with support scores of 0.66 or more (on a scale of 0 to 1) on both sides of the comparison. The support score is derived from a combination of matching exon boundaries and sequence overlap. Table S5 containing the comparison data was added as well as the following text.
(with a support score, derived from a combination of matching exon boundaries and sequence overlap, of 0.66 or more, on a scale of 0 to 1, on both sides of the comparison)

5. Based on information in Table 2, there are six sequences that align to the UMD3.1.1 assembly, but not ARS-UCD1.2. Are these six cases thought to represent bona fide deficiencies in the ARS-UCD1.2 assembly?
REPLY: It's a bit more complicated than this. The net difference in the count of sequences that do not align is 6, but there are only two transcripts that align to neither assembly. Additionally, the difference is made up by Y-linked transcripts which shouldn't be found in either assembly. I've added this to the text.
Although a greater number of transcripts failed to align to ARS-UCD1.2, this difference is made up of transcripts from Y-linked genes (Table S4). The presence of Y-linked genes in the UMD3.1.1 assembly is likely due to Y chromosome contamination from the inclusion of sequence from a bacterial artificial chromosome library prepared from Dominette's sire [34,39]. Since ARS-UCD1.2 is derived from an XX female and does not contain the Y chromosome, we recommend the inclusion of an independently assembled Y chromosome prior to analysis as is being done by the 1000 Bull Genomes Project [40].

6. In the "Improved contiguity" section, I suggest explaining to the reader the relevance of "accession prefixed with NM_ and NR_".
REPLY: Added "a manually curated set of transcript accessions" to clarify.
(a manually curated set of transcript accessions prefixed with NM_ and NR_)

7. In Table 2 the label "Number of sequences with multiple best alignments (split genes)" could be improved, as the meaning of "multiple best alignments" isn't obvious in this context.
REPLY: Changed.
Number of sequences whose best alignments span multiple loci (split genes)

8. Change last comma to period in "1,027 in ARS-UCD1.2/AR 106,"
REPLY: Fixed

9. Fix truncated sentence "to both ARS-UCD1.2 and."
REPLY: Fixed
to both ARS-UCD1.2 and UMD3.1.1.

10. It isn't clear how citations 23 and 26 will be useful, at least in their current form. Perhaps in the published article they will link to the corresponding scripts.
REPLY: Proper URLs inserted, references are now 24 and 27.
24. Bickhart, D. Scripts and documentation related to the assembly of ARS-UCD1.2: https://github.com/njdbickhart/CattleAssemblyScripts. (2019).
27. Tseng, E. Miscellaneous collection of Python and R scripts for processing Iso-Seq data: https://github.com/Magdoll/cDNA_Cupcake. (2019).

11. Regarding the UMCLK genetic map supplementary file, is the provided SQL to be used with Crimap?
REPLY: The provided SQL was used to generate the TableS2 UMCLK genetic map.csv file which is included with the manuscript, text has been added to the supplementary note to clarify.
The linkage map is stored in a PostgreSQL database at the University of Missouri. The SQL below was used to generate the TableS2_UMCLK genetic map.csv file which is included with the manuscript.
The following is a description of file TableS2_UMCLK genetic map.csv which is comma delimited with a header row. Fields are specified as below including a description of the field.

Reviewer #2:

The manuscript could be improved by addressing the following issues:
1. Was the Dovetail Chicago library constructed from DNA/chromatin from the same individual as the genome sequence data? If so, then it would be useful to confirm this. If not, then it would be useful to comment on whether this limited the accuracy of the scaffolding.
REPLY: Yes, added "from Dominette lung tissue" to clarify.
First, a Chicago library was prepared as described previously[10] from Dominette lung tissue

2. Similarly, was the optical map generated from DNA from the same individual as the genome sequence data?
REPLY: Yes, added "Dominette derived" for clarification.
Next we used the Dominette derived Bos taurus optical map BtOM1.0

3. In terms of the completeness of the assembly, did the authors detect centromeric and telomeric sequences in the chromosome assigned scaffolds?
REPLY: This is a good question. We have added this information to the "Quality assessment" section including Table S3 and Figure S1.
As a measure of the completeness of the assemblies and to define the chromosome ends, we identified centromeric[24] and telomeric[37] repeats (Table S3). For the 29 acrocentric autosomes, we identified the expected centromeric and telomeric repeats on 9 ARS-UCD1.2 chromosomes (5,6,8,10,13,14,16,17, and 18) whereas no UMD3.1.1 chromosomes contained both, mainly due to a relative lack of telomeric repeats in the assembly. ARS-UCD1.2 chromosomes 3,20, and 22 are missing both chromosome ends, while chromosomes 1,9,10, and 15 erroneously contain centromeric repeats at both ends. Finally, the metacentric X chromosome only has telomeric repeats at one end and no centromeric repeats. Telomeric repeats were only identified on UMD3.1.1 chromosome 20, centromeric repeats are found on the proper end of 22 autosomes (missing on 6,7,20,21,22,27, and 28) and the X chromosome contains centromeric repeats. All chromosomes also contain centromeric repeats dispersed throughout so it is difficult to determine if the X centromere is properly placed. Centromeric repeat regions at the start of ARS-UCD1.2 chromosome scaffolds were over 2-fold larger than their counterparts in the UMD3.1 reference (Figure S1).

4. Why was manual curation of the assembly limited to the X chromosome?
REPLY: The X chromosome was just the first to be manually curated, the entire assembly was manually curated "In all, corrections were made to chromosomes 1, 2, 5-12, 16, 18-21, 23, 26, 27, and X". We have reworded the beginning of the Methods section (c), Manual curation, to clarify.
Following gap filling, the assembly was manually curated. To start, we assessed the X chromosome using two assemblies produced from MaSuRCA[20] error-corrected reads (PacBio corrected with Illumina). The first used Canu v1.4 to assemble the MaSuRCA corrected reads and the other used Celera Assembler[17] version 8.3. MUMmer 3.0[21] alignments between these two assemblies and the gap-filled assembly were used to confirm or revise the order and orientation of X-chromosome contigs as well as place additional unplaced contigs and scaffolds. Next, the autosomal assembly structure was manually curated and oriented with an independent genetic map UMCLK (Table S2, Supplementary Note). The BLAT alignment tool[22] and BWA MEM[23] were used to map the probe and flanking sequences present on commercially available genotyping assays to identify misassemblies. Assembly gaps, Illumina read-depth coverage and alignments with dbSNP sequences and flanking sequences were used to refine breakpoints for sequence rearrangements using a combination of custom scripts in iterative fashion[24]. In all, corrections were made to chromosomes 1, 2, 5-12, 16, 18-21, 23, 26, 27, and X.

5. The second of these two sentences is a non-sequitur "Due to library size selection and loading bias, Iso-Seq is not reliable for quantitative measurements of transcript abundance. Therefore, we used a combination of public datasets and new sequenced tissues to annotate the assembly." The rationale underpinning use of other expression data (short read RNA-Seq, cDNA and ESTs) for genome annotation was presumably that the Iso-Seq data provided insufficient sequence depth to allow lowly expressed transcripts to be detected. The short read RNA-Seq, cDNA and ESTs data presumably also allowed transcripts that are restricted to other tissues, cell types, developmental stages, states and sex to be captured in the annotation.
REPLY: Agree, reworded.
Short read based RNA-seq data derived from tissues of Dominette were available in the GenBank database, as her tissues have been a freely-distributed resource for the research community. To complement and extend this data, and to ensure that the tissues used for Iso-Seq were also represented by RNA-seq data for quantitative analysis and confirmation of isoforms observed in Iso-Seq, we generated additional data avoiding overlap with existing public data.

6. What is KPH fat as sampled by the authors? KPH fat appears to be fat from kidney, pelvis and heart. Did the authors sample fat from all three of these depots and then pool them before or after preparing RNA in order to make the relevant sequence library?
REPLY: KPH fat refers to internal organ fat as opposed to subcutaneous fat. Generally speaking, and in this case, the sample is taken from the covering on the kidney capsule.
(internal organ fat taken from the covering on the kidney capsule)

7. Table 1 is poorly laid out. From the title of the Table it seems likely that the first number in each column, in which there are two numbers, refers to the whole assembly and the second to the chromosomes only. This needs to be more explicit with a footnote or legend. As the comparisons made in the text refer to the statistics for the chromosomes and the unplaced scaffolds it would be better to present these numbers in the Table rather than the statistics for the whole assembly and the chromosomes, thus requiring the reader to calculate the numbers for the unplaced scaffolds. The appearance of the Table would be improved by dividing the columns with two entries into two columns.

The appearance of this and other Tables with numbers would also be improved by right justifying the numbers.
REPLY: Table reformatted.

8. The use of separators for 1,000s and large numbers in the manuscript is inconsistent. These large numbers are much more readable with "," separators.
REPLY: Agree, fixed.

9. What was the basis for assigning and orienting scaffolds to/on chromosomes? The linkage map(s) like the sequence assemblies are agnostic about chromosome assignment and orientation on chromosomes. There is no doubt historical data linking specific genes and sequences to particular chromosome locations from cytogenetic analysis. It would be helpful to make these links explicit.
REPLY: The scaffolding section describes the use of the recombination map to scaffold the chromosomes. The UMCLK linkage map was used to orient the chromosomes. "oriented with" has been added to the text.
Finally, approximately 54k SNP markers from the bovine recombination map[12] were used to detect mis-assemblies and scaffold the 29 acrocentric autosomes.
The resulting assembly structure was then re-assessed, manually curated, and oriented with an independent genetic map UMCLK.
