# Supplementary material for: De novo assembly of the cattle reference genome with single-molecule sequencing
Source: Gigascience. 2020 Mar 19;9(3):giaa021. doi: 10.1093/gigascience/giaa021 (PMC7081964; doi:10.1093/gigascience/giaa021)
Supplement: giaa021_Supplemental_Data [file giaa021_supplemental_data.zip › SupplementalNote_UMCLK_genetic_map.docx]

**UMCLK construction**

SNP50 genotypes from over 40,000 individuals representing both beef and dairy cattle breeds were assembled into large paternal half-sib families with an average of 6,386 informative meiosis for 46,133 loci. Crimap was used to generate an initial LOD3 map (8,556 loci) followed by inclusion of additional markers based on twopoint. Local marker ordering was refined by iteratively using the flips option in 100 marker windows overlapping by 50 markers.

The linkage map is stored in a PostgreSQL database at the University of Missouri. The SQL below was used to generate the TableS2_UMCLK genetic map.csv file which is included with the manuscript.

-- Data generated at the University of Missouri

-- Robert Schnabel schnabelr@missouri.edu 08/02/2019

-- SQL to dump linkage and physical map positions

COPY (

SELECT l.snp_number, m.marker_name,

CASE WHEN lod3_marker IS NOT NULL THEN 'TRUE' ELSE 'FALSE' END AS lod3_framework_marker,

CASE WHEN lod1_marker IS NOT NULL THEN 'TRUE' ELSE 'FALSE' END AS lod1_framework_marker,

l.chr_order AS linkage_order, l.umc_chrom AS linkage_chr, l.umc_pos AS linkage_pos_cm, like_diff,

mm.chra_ars120 AS ars_ucd_chrom, mm.chr_pos_ars120 AS ars_ucd_pos,

chra_umd, chr_pos_umd

FROM markers_9913_linkage l, markers_9913_snp50 m, markers_master mm

WHERE l.snp_number = m.snp_number

AND l.snp_number = mm.snp_number

ORDER BY l.umc_chrom, l.chr_order

) TO '/scratch/schnabelr/pg_data_dump/UMC_LinkageMap_ARS-UCD_UMD3_190802.csv'

WITH CSV HEADER;

--COPY 46133 Query returned successfully in 583 msec.

The following is a description of file TableS2_UMCLK genetic map.csv which is comma delimited with a header row. Fields are specified as below including a description of the field.

snp_number [integer]: UMC internal marker ID

marker_name [character varying]: Marker name from BovineSNP50 manifest A

lod3_framework_marker [character varying]: TRUE if this marker was a framework marker at LOD=3 support, FALSE otherwise

lod1_framework_marker [character varying]: TRUE if this marker was a framework marker at LOD=1 support, FALSE otherwise

linkage_order [integer]: Order of the markers in the final map. Used to order markers with recombination fraction of zero

linkage_chr [character varying]: Chromosome number based on linkage map

linkage_pos_cm [real]: Centimorgan position based on linkage map

like_diff [real]: Difference in loglikelihood when running crimap flipsn. Negative values represent a potentially less than optimal local ordering.

ars_ucd_chrom [character varying]: ARS-UCD1.2 chromosome

ars_ucd_pos [integer]: ARS-UCD1.2 position

chra_umd [character varying]: UMD3.1 chromosome

chr_pos_umd [integer]: UMD3.1 position
