# Supplementary material for: De novo assembly of the cattle reference genome with single-molecule sequencing
Source: Gigascience. 2020 Mar 19;9(3):giaa021. doi: 10.1093/gigascience/giaa021 (PMC7081964; doi:10.1093/gigascience/giaa021)
Supplement: giaa021_Response-to-Reviewer_Comments_Original_Submission [file giaa021_response-to-reviewer_comments_original_submission.pdf]

Author's Response To Reviewer Comments

Close

Responses are also included in the Personal Cover with better formatting.

Reviewer #1:

Specific comments for revision:
1. It is not clear from section (e) of the Methods how the alignments with UniProt/SwissProtKB were generated (i.e. through BLAST, Splign, or another tool).
REPLY: Text added to the Methods section (e).
"The respective quality of the UMD3.1.1 annotation (Annotation Release 105 AR 105[33]) and AR 106 was evaluated by aligning the annotated proteins of each release to the UniProtKB/SwissProt proteins available in Entrez Protein (returned by the Entrez query srcdb_swiss_prot[properties] AND eukaryotes[orgn] on 7/29/2019) using BlastP. For each protein coding gene, the protein isoform with the best alignment based on score (or in case of a tie, based on alignment length, percent coverage or subject protein length) was chosen as the isoform representative of the gene. The counts of protein coding genes in AR 105 and AR 106 with representative isoforms covering at least 95% of the length of the UniProtKB/SwissProt proteins were then compared."

2. Related to this analysis, which release of UniProt/SwissProtKB was used?
REPLY: Date of access added to new text in Methods section (e).
"(returned by the Entrez query srcdb_swiss_prot[properties] AND eukaryotes[orgn] on 7/29/2019)"

3. Are protein sequences in the UniProt/SwissProtKB data set potentially derived in part from AR 105 or AR 106? Does this complicate interpretation of these results?
REPLY: There is a possibility that some of the proteins in the UniProt/SwissProtKB data set are from AR 105 or AR 106. However, Refseq sequences are not submitted to the International Nucleotide Sequence Database Collaboration and according to the UniProt documentation (https://www.uniprot.org/help/sequence_origin) "More than 95% of the protein sequences provided by UniProtKB come from the translations of coding sequences (CDS) submitted to the EMBL-Bank/GenBank/DDBJ nucleotide sequence resources (International Nucleotide Sequence Database Collaboration", so the chances are small. The RefSeq proteins that would be incorporated into UniProt/SwissProtKB would have been manually curated and presumably have very strong support from experimental evidence. Whether or not RefSeq proteins are part of the UniProt/SwissProtKB, the strength of the analysis relies in the difference in the number of good hits between the two annotation releases rather than in the absolute numbers of hits for each release.

4. In the 'Annotation comparison' section, the authors state that "About 2/3 of the genes (85% of protein-coding genes) are identical or nearly identical between the two datasets." What qualifies as nearly identical?
REPLY: Nearly identical genes are highly similar genes, with support scores of 0.66 or more (on a scale of 0 to 1) on both sides of the comparison. The support score is derived from a combination of matching exon boundaries and sequence overlap. Table S5 containing the comparison data was added as well as the following text.
"(with a support score, derived from a combination of matching exon boundaries and sequence overlap, of 0.66 or more, on a scale of 0 to 1, on both sides of the comparison)"

5. Based on information in Table 2, there are six sequences that align to the UMD3.1.1 assembly, but not ARS-UCD1.2. Are these six cases thought to represent bona fide deficiencies in the ARS-UCD1.2 assembly?
REPLY: It's a bit more complicated than this. The net difference in the count of sequences that do not align is 6, but there are only two transcripts that align to neither assembly. Additionally, the difference is made up by Y-linked transcripts which shouldn't be found in either assembly. I've added this to the text.
"Although a greater number of transcripts failed to align to ARS-UCD1.2, this difference is made up of

transcripts from Y-linked genes (Table S4). The presence of Y-linked genes in the UMD3.1.1 assembly is likely due to Y chromosome contamination from the inclusion of sequence from a bacterial artificial chromosome library prepared from Dominette's sire [34,39]. Since ARS-UCD1.2 is derived from an XX female and does not contain the Y chromosome, we recommend the inclusion of an independently assembled Y chromosome prior to analysis as is being done by the 1000 Bull Genomes Project [40]."

6. In the "Improved contiguity" section, I suggest explaining to the reader the relevance of "accession prefixed with NM_ and NR_".
REPLY: Added "a manually curated set of transcript accessions" to clarify.
"(a manually curated set of transcript accessions prefixed with NM_ and NR_)"

7. In Table 2 the label "Number of sequences with multiple best alignments (split genes)" could be improved, as the meaning of "multiple best alignments" isn't obvious in this context.
REPLY: Changed.
"Number of sequences whose best alignments span multiple loci (split genes)"

8. Change last comma to period in "1,027 in ARS-UCD1.2/AR 106,"
REPLY: Fixed

9. Fix truncated sentence "to both ARS-UCD1.2 and."
REPLY: Fixed
"to both ARS-UCD1.2 and UMD3.1.1."

10. It isn't clear how citations 23 and 26 will be useful, at least in their current form. Perhaps in the published article they will link to the corresponding scripts.
REPLY: Proper URLs inserted, references are now 24 and 27.
"24. Bickhart, D. Scripts and documentation related to the assembly of ARS-UCD1.2: https://github.com/njdbickhart/CattleAssemblyScripts. (2019)."
"27. Tseng, E. Miscellaneous collection of Python and R scripts for processing Iso-Seq data: https://github.com/Magdoll/cDNA_Cupcake. (2019)."

11. Regarding the UMCLK genetic map supplementary file, is the provided SQL to be used with Crimap?
REPLY: The provided SQL was used to generate the TableS2 UMCLK genetic map.csv file which is included with the manuscript, text has been added to the supplementary note to clarify.
"The linkage map is stored in a PostgreSQL database at the University of Missouri. The SQL below was used to generate the TableS2_UMCLK genetic map.csv file which is included with the manuscript."
"The following is a description of file TableS2_UMCLK genetic map.csv which is comma delimited with a header row. Fields are specified as below including a description of the field."

Reviewer #2:

The manuscript could be improved by addressing the following issues:
1. Was the Dovetail Chicago library constructed from DNA/chromatin from the same individual as the genome sequence data? If so, then it would be useful to confirm this. If not, then it would be useful to comment on whether this limited the accuracy of the scaffolding.
REPLY: Yes, added "from Dominette lung tissue" to clarify.
"First, a Chicago library was prepared as described previously[10] from Dominette lung tissue"

2. Similarly, was the optical map generated from DNA from the same individual as the genome sequence data?
REPLY: Yes, added "Dominette derived" for clarification.
"Next we used the Dominette derived Bos taurus optical map BtOM1.0"

3. In terms of the completeness of the assembly, did the authors detect centromeric and telomeric sequences in the chromosome assigned scaffolds?
REPLY: This is a good question. We have added this information to the "Quality assessment" section including Table S3 and Figure S1.
"As a measure of the completeness of the assemblies and to define the chromosome ends, we identified centromeric[24] and telomeric[37] repeats (Table S3). For the 29 acrocentric autosomes, we identified the expected centromeric and telomeric repeats on 9 ARS-UCD1.2 chromosomes (5,6,8,10,13,14,16,17, and 18) whereas no UMD3.1.1 chromosomes contained both, mainly due to a relative lack of telomeric repeats in the assembly. ARS-UCD1.2 chromosomes 3,20, and 22 are missing both chromosome ends,

while chromosomes 1,9,10, and 15 erroneously contain centromeric repeats at both ends. Finally, the metacentric X chromosome only has telomeric repeats at one end and no centromeric repeats. Telomeric repeats were only identified on UMD3.1.1 chromosome 20, centromeric repeats are found on the proper end of 22 autosomes (missing on 6,7,20,21,22,27, and 28) and the X chromosome contains centromeric repeats. All chromosomes also contain centromeric repeats dispersed throughout so it is difficult to determine if the X centromere is properly placed. Centromeric repeat regions at the start of ARS-UCD1.2 chromosome scaffolds were over 2-fold larger than their counterparts in the UMD3.1 reference (Figure S1)."

4. Why was manual curation of the assembly limited to the X chromosome?
REPLY: The X chromosome was just the first to be manually curated, the entire assembly was manually curated "In all, corrections were made to chromosomes 1, 2, 5-12, 16, 18-21, 23, 26, 27, and X". We have reworded the beginning of the Methods section (c), Manual curation, to clarify.
"Following gap filling, the assembly was manually curated. To start, we assessed the X chromosome using two assemblies produced from MaSuRCA[20] error-corrected reads (PacBio corrected with Illumina). The first used Canu v1.4 to assemble the MaSuRCA corrected reads and the other used Celera Assembler[17] version 8.3. MUMmer 3.0[21] alignments between these two assemblies and the gap-filled assembly were used to confirm or revise the order and orientation of X-chromosome contigs as well as place additional unplaced contigs and scaffolds. Next, the autosomal assembly structure was manually curated and oriented with an independent genetic map UMCLK (Table S2, Supplementary Note). The BLAT alignment tool[22] and BWA MEM[23] were used to map the probe and flanking sequences present on commercially available genotyping assays to identify misassemblies. Assembly gaps, Illumina read-depth coverage and alignments with dbSNP sequences and flanking sequences were used to refine breakpoints for sequence rearrangements using a combination of custom scripts in iterative fashion[24]. In all, corrections were made to chromosomes 1, 2, 5-12, 16, 18-21, 23, 26, 27, and X."

5. The second of these two sentences is a non-sequitur "Due to library size selection and loading bias, Iso-Seq is not reliable for quantitative measurements of transcript abundance. Therefore, we used a combination of public datasets and new sequenced tissues to annotate the assembly." The rationale underpinning use of other expression data (short read RNA-Seq, cDNA and ESTs) for genome annotation was presumably that the Iso-Seq data provided insufficient sequence depth to allow lowly expressed transcripts to be detected. The short read RNA-Seq, cDNA and ESTs data presumably also allowed transcripts that are restricted to other tissues, cell types, developmental stages, states and sex to be captured in the annotation.
REPLY: Agree, reworded.
"Short read based RNA-seq data derived from tissues of Dominette were available in the GenBank database, as her tissues have been a freely-distributed resource for the research community. To complement and extend this data, and to ensure that the tissues used for Iso-Seq were also represented by RNA-seq data for quantitative analysis and confirmation of isoforms observed in Iso-Seq, we generated additional data avoiding overlap with existing public data."

6. What is KPH fat as sampled by the authors? KPH fat appears to be fat from kidney, pelvis and heart. Did the authors sample fat from all three of these depots and then pool them before or after preparing RNA in order to make the relevant sequence library?
REPLY: KPH fat refers to internal organ fat as opposed to subcutaneous fat. Generally speaking, and in this case, the sample is taken from the covering on the kidney capsule.
"(internal organ fat taken from the covering on the kidney capsule)"

7. Table 1 is poorly laid out. From the title of the Table it seems likely that the first number in each column, in which there are two numbers, refers to the whole assembly and the second to the chromosomes only. This needs to be more explicit with a footnote or legend. As the comparisons made in the text refer to the statistics for the chromosomes and the unplaced scaffolds it would be better to present these numbers in the Table rather than the statistics for the whole assembly and the chromosomes, thus requiring the reader to calculate the numbers for the unplaced scaffolds. The appearance of the Table would be improved by dividing the columns with two entries into two columns. The appearance of this and other Tables with numbers would also be improved by right justifying the numbers.
REPLY: Table reformatted.

8. The use of separators for 1,000s and large numbers in the manuscript is inconsistent. These large numbers are much more readable with "," separators.

REPLY: Agree, fixed.

9. What was the basis for assigning and orienting scaffolds to/on chromosomes? The linkage map(s) like the sequence assemblies are agnostic about chromosome assignment and orientation on chromosomes. There is no doubt historical data linking specific genes and sequences to particular chromosome locations from cytogenetic analysis. It would be helpful to make these links explicit.
REPLY: The scaffolding section describes the use of the recombination map to scaffold the chromosomes. The UMCLK linkage map was used to orient the chromosomes. "oriented with" has been added to the text.
"Finally, approximately 54k SNP markers from the bovine recombination map[12] were used to detect mis-assemblies and scaffold the 29 acrocentric autosomes."
"The resulting assembly structure was then re-assessed, manually curated, and oriented with an independent genetic map UMCLK."

Close
