# Supplementary material for: De novo assembly of the cattle reference genome with single-molecule sequencing
Source: Gigascience. 2020 Mar 19;9(3):giaa021. doi: 10.1093/gigascience/giaa021 (PMC7081964; doi:10.1093/gigascience/giaa021)
Supplement: giaa021_Reviewer_1_Report_Original_Submission — Paul Stothard -- 10/7/2019 Reviewed [file giaa021_reviewer_1_report_original_submission.pdf]

Reviewer Report

Title: De novo assembly of the cattle reference genome with single-molecule sequencing

Version: Original Submission **Date:** 10/7/2019

Reviewer name: Paul Stothard

Reviewer Comments to Author:

The authors present a superior Bos taurus genome assembly, generated by a combination of long and short read sequencing and up-to-date methods for de novo assembly and scaffolding. They also generated an improved transcriptome by combining publicly available RNA-Seq short read data with both long and short reads that they generated from RNA extracted from 22 tissues. Multiple independent methods and datasets were frequently used to refine and assess the quality of the genomic assembly and transcripts. The methods used in this manuscript are appropriate given the aims of the study.

The authors' conclusions about the quality of their genome assembly relative to the reference assembly UMD3.1.1 are well-supported by the data. Both assemblies were evaluated using a short-read dataset that was not used for refinement of the ARS-UCD1.2 assembly, with ARS-UCD1.2 showing a clear improvement in quality scores. The authors also show improved RefSeq transcript mapping to the new assembly, and better alignment of ARS-UCD1.2-derived proteins with those found in the UniProt database.

Specific comments for revision:

1. It is not clear from section (e) of the Methods how the alignments with UniProt/SwissProtKB were generated (i.e. through BLAST, Splign, or another tool).
2. Related to this analysis, which release of UniProt/SwissProtKB was used?
3. Are protein sequences in the UniProt/SwissProtKB data set potentially derived in part from AR 105 or AR 106? Does this complicate interpretation of these results?
4. In the 'Annotation comparison' section, the authors state that "About 2/3 of the genes (85% of protein-coding genes) are identical or nearly identical between the two datasets." What qualifies as nearly identical?
5. Based on information in Table 2, there are six sequences that align to the UMD3.1.1 assembly, but not ARS-UCD1.2. Are these six cases thought to represent bona fide deficiencies in the ARS-UCD1.2 assembly?
6. In the "Improved contiguity" section, I suggest explaining to the reader the relevance of "accession prefixed with NM_ and NR_".
7. In Table 2 the label "Number of sequences with multiple best alignments (split genes)" could be improved, as the meaning of "multiple best alignments" isn't obvious in this context.
8. Change last comma to period in "1,027 in ARS-UCD1.2/AR 106,"
9. Fix truncated sentence "to both ARS-UCD1.2 and."
10. It isn't clear how citations 23 and 26 will be useful, at least in their current form. Perhaps in the published article they will link to the corresponding scripts.

11. Regarding the UMCLK genetic map supplementary file, is the provided SQL to be used with Crimap?

Level of Interest

Please indicate how interesting you found the manuscript: Choose an item.

Quality of Written English

Please indicate the quality of language in the manuscript: Choose an item.

Declaration of Competing Interests

Please complete a declaration of competing interests, considering the following questions:

- Have you in the past five years received reimbursements, fees, funding, or salary from an organisation that may in any way gain or lose financially from the publication of this manuscript, either now or in the future?
- Do you hold any stocks or shares in an organisation that may in any way gain or lose financially from the publication of this manuscript, either now or in the future?
- Do you hold or are you currently applying for any patents relating to the content of the manuscript?
- Have you received reimbursements, fees, funding, or salary from an organization that holds or has applied for patents relating to the content of the manuscript?
- Do you have any other financial competing interests?
- Do you have any non-financial competing interests in relation to this paper?

If you can answer no to all of the above, write 'I declare that I have no competing interests' below. If your reply is yes to any, please give details below.

I declare that I have no competing interests

I agree to the open peer review policy of the journal. I understand that my name will be included on my report to the authors and, if the manuscript is accepted for publication, my named report including any attachments I upload will be posted on the website along with the authors' responses. I agree for my report to be made available under an Open Access Creative Commons CC-BY license (http://creativecommons.org/licenses/by/4.0/). I understand that any comments which I do not wish to be included in my named report can be included as confidential comments to the editors, which will not be published.

Choose an item.

To further support our reviewers, we have joined with Publons, where you can gain additional credit to further highlight your hard work (see: https://publons.com/journal/530/gigascience). On publication of this paper, your review will be automatically added to Publons, you can then choose whether or not to claim your Publons credit. I understand this statement.

Yes Choose an item.
