# Supplementary material for: De novo assembly of the cattle reference genome with single-molecule sequencing
Source: Gigascience. 2020 Mar 19;9(3):giaa021. doi: 10.1093/gigascience/giaa021 (PMC7081964; doi:10.1093/gigascience/giaa021)
Supplement: giaa021_Reviewer_2_Report_Original_Submission — Alan Archibald -- 10/22/2019 Reviewed [file giaa021_reviewer_2_report_original_submission.pdf]

Reviewer Report

Title: De novo assembly of the cattle reference genome with single-molecule sequencing

Version: Original Submission **Date:** 10/22/2019

Reviewer name: Alan Archibald

Reviewer Comments to Author:

The authors present a concise description of the generation and analysis of a new and improved reference genome sequence for cattle (Bos taurus).
The authors have undertaken multiple quality checks on this new genome assembly (ARS-UCD1.2) and convincingly demonstrate that it represents a significant improvement on the earlier reference assemblies.
The sequence data are freely accessible in the public sequence databases in accordance with the data sharing norms of the worldwide genomics research community.
Annotated versions of this new cattle reference genome sequence are available in the NCBI, Ensembl and UCSC genome browsers.
The manuscript could be improved by addressing the following issues:
1. Was the Dovetail Chicago library constructed from DNA/chromatin from the same individual as the genome sequence data? If so, then it would be useful to confirm this. If not, then it would be useful to comment on whether this limited the accuracy of the scaffolding.
2. Similarly, was the optical map generated from DNA from the same individual as the genome sequence data?
3. In terms of the completeness of the assembly, did the authors detect centromeric and telomeric sequences in the chromosome assigned scaffolds?
4. Why was manual curation of the assembly limited to the X chromosome?
5. The second of these two sentences is a non-sequitur "Due to library size selection and loading bias, Iso-Seq is not reliable for quantitative measurements of transcript abundance. Therefore, we used a combination of public datasets and new sequenced tissues to annotate the assembly." The rationale underpinning use of other expression data (short read RNA-Seq, cDNA and ESTs) for genome annotation was presumably that the Iso-Seq data provided insufficient sequence depth to allow lowly expressed transcripts to be detected. The short read RNA-Seq, cDNA and ESTs data presumably also allowed transcripts that are restricted to other tissues, cell types, developmental stages, states and sex to be captured in the annotation.
6. What is KPH fat as sampled by the authors? KPH fat appears to be fat from kidney, pelvis and heart. Did the authors sample fat from all three of these depots and then pool them before or after preparing RNA in order to make the relevant sequence library?
7. Table 1 is poorly laid out. From the title of the Table it seems likely that the first number in each column, in which there are two numbers, refers to the whole assembly and the second to the chromosomes only. This needs to be more explicit with a footnote or legend. As the comparisons made in the text refer to the statistics for the chromosomes and the unplaced scaffolds it would be better to

present these numbers in the Table rather than the statistics for the whole assembly and the chromosomes, thus requiring the reader to calculate the numbers for the unplaced scaffolds. The appearance of the Table would be improved by dividing the columns with two entries into two columns. The appearance of this and other Tables with numbers would also be improved by right justifying the numbers.

8. The use of separators for 1,000s and large numbers in the manuscript is inconsistent. These large numbers are much more readable with "," separators.

9. What was the basis for assigning and orienting scaffolds to/on chromosomes? The linkage map(s) like the sequence assemblies are agnostic about chromosome assignment and orientation on chromosomes. There is no doubt historical data linking specific genes and sequences to particular chromosome locations from cytogenetic analysis. It would be helpful to make these links explicit.

Level of Interest

Please indicate how interesting you found the manuscript: Choose an item.

Quality of Written English

Please indicate the quality of language in the manuscript: Choose an item.

Declaration of Competing Interests

Please complete a declaration of competing interests, considering the following questions:

- Have you in the past five years received reimbursements, fees, funding, or salary from an organisation that may in any way gain or lose financially from the publication of this manuscript, either now or in the future?
- Do you hold any stocks or shares in an organisation that may in any way gain or lose financially from the publication of this manuscript, either now or in the future?
- Do you hold or are you currently applying for any patents relating to the content of the manuscript?
- Have you received reimbursements, fees, funding, or salary from an organization that holds or has applied for patents relating to the content of the manuscript?
- Do you have any other financial competing interests?
- Do you have any non-financial competing interests in relation to this paper?

If you can answer no to all of the above, write 'I declare that I have no competing interests' below. If your reply is yes to any, please give details below.

I declare that I have no competing interests

I agree to the open peer review policy of the journal. I understand that my name will be included on my report to the authors and, if the manuscript is accepted for publication, my named report including any attachments I upload will be posted on the website along with the authors' responses. I agree for my report to be made available under an Open Access Creative Commons CC-BY license (http://creativecommons.org/licenses/by/4.0/). I understand that any comments which I do not wish to be included in my named report can be included as confidential comments to the editors, which will not be published.

Choose an item.

To further support our reviewers, we have joined with Publons, where you can gain additional credit to further highlight your hard work (see: https://publons.com/journal/530/gigascience). On publication of this paper, your review will be automatically added to Publons, you can then choose whether or not to claim your Publons credit. I understand this statement.

Yes Choose an item.
